# Relationship Between Fruit and Vegetables Intake and Common Mental Disorders in Youth: A Systematic Review

**DOI:** 10.3389/phrs.2022.1604686

**Published:** 2022-09-20

**Authors:** Julia Dabravolskaj, Shelby Marozoff, Katerina Maximova, Sandra Campbell, Paul J. Veugelers

**Affiliations:** ^1^ School of Public Health, University of Alberta, Edmonton, AB, Canada; ^2^ MAP Centre for Urban Health Solutions, Li Ka Shing Knowledge Institute, St. Michael’s Hospital, Toronto, ON, Canada; ^3^ Dalla Lana School of Public Health, University of Toronto, Toronto, ON, Canada; ^4^ John W. Scott Health Sciences Library, Faculty of Medicine and Dentistry, University of Alberta, Edmonton, AB, Canada

**Keywords:** adolescents, youth, mental health and wellbeing, common mental disorders, healthy diet, vegetables and fruit, depression, anxiety

## Abstract

**Objective:** Recent evidence suggests that adequate fruit and vegetables intake (FVI) might be associated with lower risk of common mental disorders (CMDs) in adults, but studies in youth are also beginning to emerge and are synthesized in this systematic review.

**Methods:** Online databases were searched from inception to 30 October 2020 to locate cross-sectional, cohort, and case-control studies focusing on the FVI and CMDs in youth (i.e., 10–18 years old). The risk of bias of studies was assessed using Joanna Briggs Institute Critical Appraisal Tool and the Newcastle-Ottawa quality assessment scale.

**Results:** Among 3,944 records identified, 12 studies (8 cross-sectional, 1 case-control, and 3 prospective cohort studies) were included in the final synthesis. None of the prospective cohort studies identified a statistically significant association between FVI and CMDs in youth, although inconsistent associations were reported in cross-sectional and case-control studies.

**Conclusion:** The lack of associations between FVI and CMDs in youth, along with consistent associations in adults, might be explained by the accumulation of risk theoretical model and methodological challenges.

## Introduction

Mental disorders constitute a significant global public health burden [1–3] and result from an intricate interplay of multiple factors. This interplay is particularly potent during adolescence (i.e., 10–19 years old) when more than three-quarters of all life-time mental disorders, especially common mental disorders (CMDs) such as depression and anxiety, manifest for the first time [4, 5]. In fact, the cumulative probability of CMDs rises steeply from around 5% in early adolescence to as high as 20% by the end of adolescence [6]. Mental disorders in adolescence are associated with many long-term negative psychosocial outcomes, including low educational attainment, poor work performance [7], difficulty developing stable relationships and social networks, unemployment [8], as well as future mental disorders [9], substance abuse, and suicide [6]. Often underdiagnosed [4], they can be difficult to manage due to limited effectiveness of available treatment options [10] and high rates of recurrence [6] and comorbidity [11].

With one in five adolescents living with mental disorders worldwide [12], there is a recognized need for the development and implementation of effective primary prevention strategies [13]. Recent systematic reviews [14, 15] of observational studies point to the association between adherence to high quality diet (i.e., rich in fruit, vegetables, legumes, nuts and whole grains [16]) and lower incidence of CMDs in a general population of youth. However, diet quality is often conceptualized and measured differently, making it difficult to compare results across different studies. To circumvent this challenge, fruit and vegetables intake (FVI) is often used as a simple indicator of overall diet quality [17].

A systematic review [18] of 16 cross-sectional, 9 cohort, and 2 case-control on the association between FVI and mental disorders in adults showed that the highest category of FVI was associated with up to 17% lower risk of depression in cohort studies, with higher magnitudes of the associations (i.e., up to 25% lower risk of depression) observed in cross-sectional studies. Moreover, every 100-g increase in fruit and vegetable intake was associated with a 3% reduced risk of depression in cohort studies. However, to our knowledge, evidence on this association in adolescents has not yet been synthesized. This paper fills in this gap, with particular attention given to the methodological aspects of available studies to inform future research in the field of nutritional psychiatry in youth.

## Methods

### Search Strategy

A medical librarian [SC (Sandra Campbell)] searched the following databases: Prospero, Wiley Cochrane Library, Ovid Embase, Ovid Medline, Ovid PsycInfo, EBSCO CINAHL, ProQuest Dissertations and Theses Global, Food Science and Technology Abstracts (WOS) and CAB Abstracts (WOS). Each of the databases was searched from inception to 30 October 2020. The search strategy included both text words and controlled vocabulary (e.g., MeSH, EMTREE, etc.) for the terms “fruits or vegetables” and “anxiety or depression” (see Supplementary File S1). Studies limited to adults and very young children were excluded. In addition, bibliographies of relevant studies and researcher-identified databases were hand searched. All identified records were exported to Covidence systematic review software [19], and duplicates were automatically removed (see PRISMA flowchart, Supplementary File S2). Language restriction was not applied; when necessary, a native language speaker was identified in the research community at the University of Alberta and asked to translate the paper, assess eligibility of the study and extract data. This systematic review was registered on PROSPERO (CRD42020148625, 1 August 2020).

### Inclusion Criteria

Two reviewers [JD and SM] independently reviewed the titles and abstracts on the Covidence platform [19]. [JD and SM] documented and compared reasons for exclusion. Bibliographies of included papers were reviewed for relevant papers independently by [JD and SM]. Disagreements during the screening process were resolved by consensus. We included observational (i.e., cohort, case-control, and cross-sectional) studies that focused on FVI, measured combined or separately, and CMDs in community-dwelling adolescents (see detailed inclusion and exclusion criteria in Table 1). Inclusion of primary studies was not limited by sex, ethnicity, or any socioeconomic determinants of health. Studies that included adolescents but also extended outside the specified age range (i.e., 10–18 years old) were assessed on a case-by-case basis as to whether they could meaningfully contribute to the systematic review. If data were duplicated in more than one study, only the latest study or the study with the largest sample size was considered. Studies that did not report any estimates of the association of interest and did not provide any data that could be used to calculate any measures of association were excluded.

**TABLE 1:** Inclusion and exclusion criteria (systematic review, all countries, up to 2020).

	Inclusion criteria	Exclusion criteria
Population	Community-dwelling 10–18 years old adolescents; general population if the association was studied in adolescents as part of subgroups analyses	Non-human subjects; institutionalized adolescents; studies focusing solely on children (<10 years old) or adults (≥19 years old)
Exposure of interest	FVI measured in terms of frequency of consumption or servings or grams per day (80 g was considered one serving, World Health Organization, 2004 [22])	Other diet constructs (e.g., eating behaviors) considered alone, rather than in combination with FVI.
Outcome of interest	Common mental disorders (i.e., depression, anxiety, or co-morbid depression and anxiety), as diagnosed by physicians, using validated tools, or self-reporting	Other mental disorders (including those with anxiety and/or depressive components, eating disorders, psychological distress, attention deficit hyperactivity disorder). For studies where outcomes were measured with a single question (as opposed to a validated scale), reviewers assessed if the question explicitly stated or implied mental disorders other than the outcome of interest

### Data Extraction and Management

Upon finalizing the list of included studies, [JD and SM] independently extracted the following data: study details (first author, title, publication year, journal, objectives of the study, study design, follow-up years, study duration, recruitment procedures utilized, description of the exposure(s) and exposure assessment tools, comparator, description of the outcome(s) and outcome assessment tools, sample size, mean age or age range at baseline, sex of participants included in the study); analysis and results (i.e., statistical methods used to produce the measure and magnitude of association, standard error, standard deviation for the exposure and control groups, 95% CI, *p*-value, confounders adjusted for in the analysis); and author conclusions. If data in a selected study was missing or lacked sufficient details, [JD] contacted corresponding authors for additional information. Where the results of several models where presented, data were extracted for all models. Study-specific methods and results (e.g., statistical methods used, comparators, effect measures) are presented in Supplementary Table S1 (Supplementary File S3).

### Risk of Bias Assessment

The Newcastle-Ottawa quality assessment scale [20] was used to assess the quality of cohort and case-control studies. The Joanna Briggs Institute (JBI) Critical Appraisal Tool for cross-sectional studies [21] was used to assess the quality of cross-sectional studies. One question was excluded from this tool (i.e., “Were objective, standard criteria used for measurement of the condition?”), since the focus was on the general population rather than specific diagnostic methods or clinical populations. As part of the quality assessment process, we assessed whether important confounding factors [particularly, socioeconomic status (SES) which can affect both person’s diet and mental health outcomes] were identified and adjusted for in the included studies. Discrepancies resulting from the independent application of quality assessment tools by [JD and SM] were resolved by consensus. Since the systematic review aimed to map the existing literature and highlight methodological challenges and areas for further research, studies were not excluded based on risk of bias assessment. The utility of introducing a qualitative score has long been discredited [33]; instead, quality assessments of the selected cross-sectional, cohort and case-control studies are summarized in Tables 2–4, respectively.

**TABLE 2:** Quality assessment of cross-sectional studies included in the systematic review (systematic review, all countries, up to 2020).

Cross-sectional studies	Were the criteria for inclusion in the sample clearly defined?	Were the study subjects and the setting described in detail?	Was the exposure measured in a valid and reliable way?	Were confounding factors identified?	Were strategies to deal with confounding factors stated?	Were the outcomes measured in a valid and reliable way?	Was appropriate statistical analysis used?
Arat, 2017 [23]	No	No	Yes	Yes	Yes	No	Yes
Arat, 2015 [24]	No	No	Yes	No	No	No	No
Hoare et al., 2019 [25]	No	Yes	Yes	Yes	Yes	Yes	Yes
Hoare et al., 2014 [26]	No	Yes	Yes	Yes	Yes	Yes	Yes
Hoare et al., 2018 [27]	No	Yes	Yes	Yes	Yes	Yes	Yes
Park et al., 2018 [31]	No	Yes	Yes	Yes	Yes	Yes	Yes
Hong and Peltzer, 2017 [28]	No	Yes	Yes	Yes	Yes	No	Yes
Liu et al., 2020 [29]	Yes	Yes	Yes	Yes	Yes	No	Yes
Park et al., 2018 [30]	Yes	Yes	Yes	Yes	Yes	No	Yes
Winpenny et al., 2018 [32]	No	Yes	Yes	Yes	Yes	Yes	Yes

**TABLE 3:** Quality assessment of cohort studies included in the systematic review (systematic review, all countries, up to 2020).

Cohort studies	Representativeness of the exposed cohort: A: truly representative of the average (describe) in the community; B: somewhat representative of the average in the community; C: selected group of users; D: no description of the derivation of the cohort	Selection of the non exposed cohort: A: drawn from the same community as the exposed cohort; B: drawn from a different source; C: no description of the derivation of the non-exposed cohort	Ascertainment of exposure: A: secure record; B: structured interview; C: written self report; D: no description	Demonstration that outcome of interest was not present at start of study: A: yes; B: no.	Comparability of cohorts on the basis of the design or analysis: A: study controls for socioeconomic status; B: study controls for any additional factor	Assessment of outcome: A: independent blind assessment; B: record linkage; C: self report; D: no description	Was follow-up long enough for outcomes to occur: A: yes; B: no	Adequacy of follow up of cohorts: A: complete follow up - all subjects accounted for; B: subjects lost to follow up unlikely to introduce bias (≥70% follow-up) or description provided of those lost; C: follow up rate <70% and no description of those lost; D: no statement
Hoare et al., 2016 [31]	B	A	C	A	B	C	A (2 years)	B
McMartin et al., 2012 [34]	A	A	C	A	A and B	B	A (3 years)	A
Winpenny et al., 2018 [32]	B	A	C	A	A and B	C	A (3 years)	C

**TABLE 4:** Quality assessment of case-control studies included in the systematic review (systematic review, all countries, up to 2020).

Case-control studies	Is the case definition adequate: A: yes, with independent validation; B: yes (e.g., record linkage, self reports); C: no description	Representativeness of the cases: A: consecutive or obviously representative series of cases; B: potential for selection biases or not stated	Selection of controls: A: community controls; B: hospital controls; C: no description	Definition of controls: A: no history of disease (endpoint); B: no description of source	Comparability of cases and controls on the basis of the design or analysis: A: study control for socioeconomic status; B: study controls for additional factors	Ascertainment of exposure (A: secure record (e.g., surgical records); B: structured interview blinded to case/control status; C: interview not blinded to case/control status; D: written self report or medical record only; E: no description	Same method of ascertainment for cases and control: A: yes; B: no	Non-response rate: A: same rate for both groups; B: non respondents described; C: rate different and no designation
Kim et al., 2015 [35]	B	B	A	A	B	D	A	C

### Data Analysis and Reporting

Considering substantial heterogeneity between studies in terms of study design, exposure and outcome definition and assessment methods, included covariates, measures of association, and results of the risk of bias assessment, the authors refrained from pooling data in a meta-analysis. Moreover, the number of studies available was not sufficient to pool in sub-group analyses. Therefore, a narrative synthesis is provided. The review follows Preferred Reporting Items for Systematic Reviews and Meta-Analyses (PRISMA) reporting guidelines (Supplementary File S4).

## Results

The search yielded 3,944 records to assess for eligibility, 903 duplicates were removed. Among 117 records that were examined in full, 12 studies were included in the final analysis. The articles were published between 2012 and 2020 and included analysis of data collected in United States, United Kingdom, Greece, Australia, Canada, South Korea, Saint Lucia, Egypt, Saint Vincent and the Grenadines, Djibouti, Morocco, Myanmar, Zambia, Tanzania, Venezuela, Grenada, Lebanon, China, Indonesia, Thailand, Uganda, Tunisia, Botswana, Sri Lanka, India, Seychelles, Guyana, Ecuador, Jordan, Argentina, and Kenya. Table 5 and Supplementary Table S1 (see Supplementary File S4) provide detailed information on the study characteristics and results of the included studies, respectively. Eight studies [23–27, 28–30] used cross-sectional design, three studies [31, 32, 34] were prospective cohort studies (two [31, 32] included analyses of both cross-sectional and longitudinal data), and one [35] was a case-control study. Sample sizes ranged from 603 [32] to 65,528 [28] adolescents in cross-sectional studies, from 472 [31] to 3,757 [34] in cohort studies, and 849 [35] in the case-control study. Except for one study that focused exclusively on female youth [35], all other studies included approximately equal numbers of male and female youth. Age distribution was within the prespecified age limits (i.e., 10–18 years old) in all but one study [25] that included a range of 9–13 years old children attending Grade 5 and 6, with 58% of the sample being older children (11–13 years old).

**TABLE 5:** Description of studies included in the systematic review (systematic review, all countries, up to 2020).

Author(s) and publication year	Study design	Country	Sample size	Mean age or age range (at baseline if a cohort study)	%females	Follow-up years	Exposure(s)	Outcome(s)	Comments and conclusions
Arat, 2017 [23]	Cross-sectional	Botswana	2,197	11–17 years old	55%	N/A	F, V^a^: Single-item dietary measure as part of the GSHS “During the past 30 days, how many times per day did you usually eat fruit?” and “During the past 30 days, how many times per day did you usually eat vegetable?”	Depression and anxiety assessed by single questions: “During the past 12 months, did you ever feel so sad or hopeless almost every day for 2 weeks or more in a row that you stopped doing your usual activities?” and “During the past 12 months, how often have you been so worried about something that you could not sleep at night?”	“… higher fruit intake as a risk factor for depression, anxiety (except the United Republic of Tanzania)… higher vegetable consumption as a risk factor for depression, anxiety (except the United Republic of Tanzania and Zambia)…”
		Kenya	3,691		51.3%				
		Seychelles	1,432		52.2%				
		Uganda	3,215		48.8%				
		Tanzania	2,176		52.1%				
		Zambia	2,257		51.1%				
Arat, 2015 [24]	Cross-sectional	United States	10,563	12–18 years old	Asian American 52.2%, African American 49.6%, Caucasian 47.7%	N/A	F, V: Single-item dietary measure as part of the YRBS^b^ “During the past 7 days, how many times did you eat fruit?”); “During the past 7 days, how many times did you eat other vegetables? (Do not count green salad, potatoes, or carrots.)”	Depression assessed by a single question: “During the past 12 months, did you ever feel so sad or hopeless almost every day for 2 weeks or more in a row that you stopped doing some usual activities?”	No association between F and V intake and depression; however, causal language throughout the article (e.g. “risk factors for depression specific to Asians, and not Caucasians or Africans, was lower carrot consumption”)
Hoare et al., 2019 [25]	Cross-sectional	Greece	2,240	9–13 years old	50%	N/A	F, V: 24-h recall morning interviews conducted by trained dietitians and nutritionists on 2 consecutive weekdays and 1 weekend day	Emotional functioning (i.e., depression) assessed by COOPS/WONCA questionnaire: “During the past 2 weeks, how much were you pre-occupied with emotional problems such as feeling anxious, depressed, irritable or downhearted and sad?”	“There were no association observed between the consumption of fruits and vegetables and emotional functioning”
Hoare et al., 2014 [26]	Cross-sectional	Australia	800	11.8–14.9 years old	55%	N/A	FV: single item dietary measure as part of the ABAKQ^c^ “How many servings of fruit/vegetables they consumed on the last school day, including those eaten at home?”	Depression assessed by the SMFQ	“neither fruit and vegetable nor takeaway food consumption were related to depressive symptomatology in multivariate analyses.”
Hoare et al., 2018 [27]	Cross-sectional^d^	United States	3,696	15.9 (1.7)	Both males and females were included in the sample, but %females not reported	N/A	F, V: Single-item dietary measure “How often did you eat fruit or drink fruit juice yesterday?” and the same question for vegetable consumption	Depression assessed by the 20-item CES-D	“Fruit consumption was cross-sectionally related to reduced odds of depression in adolescence in both males and females, both before and after controlling for covariates. Vegetable consumption among females was cross-sectionally associated with reduced odds of depression in adolescence”
Hoare et al., 2016 [31]	1) Cross-sectional and 2) prospective cohort	Australia	634	13.1 (0.6)	53.3%	Wave 1 (May 2012), Wave 2 (May 2014)	FV: “How many servings of fruit/ vegetables they consumed on the last school day, including those eaten at home?” as part of the ABAKQ	Depression assessed by the SMFQ	FVI was not a significant predictor in univariate analysis, hence not entered in further models and not commented on
Hong and Peltzer, 2017 [28]	Cross-sectional	Korea	65,528	12–18 years old (mean age 15.1)	47.8%	N/A	F, V: single-item dietary measure as part of KYRBS^e^ Participants were asked about the frequency of fruits (excluding fruit juices) and vegetable dishes (excluding Kimchi) over the past 7 days	Depression symptoms assessed by a single question: “Have you experienced sadness or despair to the degree that you stopped your daily routine for the recent 12 months?”	“Positive dietary behaviours (fruit and vegetable consumption … ) were negatively associated with perceived stress and depression symptoms”
Kim et al., 2015 [35]	Case-control	Korea	849	15 (1.5)	100%	N/A (depressive symptoms were assessed during recruitment, while data on dietary patterns was obtained by FFQ in the past 12 months)	F, V: FFQ for the KYRBS; frequency range of the FFQ items in the past 12 months was classified into nine categories (never or seldom, once per month, 2–3 times per month, once per week, 2–4 times per week, 5–6 times per week, once per day, twice per day and three times per day) and the portion size was divided into three categories (small, medium and large)	Depression assessed by the Korean version of the Beck Depression Inventory	“…consumption of green vegetables and 1 to 3 servings/day of fruits was associated with decreased risk of depression”
Liu et al., 2020 [29]	Cross-sectional	25 low- and middle-income countries^f^, see below	65,267	12–15 years old	Country-specific, ranging between 40.5% and 57.9%	N/A	F, V, FV: Single-item measure as part of the GSHS^g^ “During the past 30 days, how many times per day did you usually eat fruit, such as apples, bananas, oranges?” and “During the past 30 days, how many times per day did you usually eat vegetables, such as salads, spinach, eggplant, tomatoes, and cucumbers?”	Depressive and anxiety symptoms assessed by a single question: “During the past 12 months, did you ever feel so sad or hopeless almost every day for 2 weeks or more in a row that you stopped doing your usual activities?” and “During the past 12 months, how often have you been so worried about something that you could not sleep at night?”	When country-specific estimates were combined in a meta-analysis, inadequate vs. adequate FVI was associated with a higher risk of depressive symptoms but not anxiety symptoms”
		Saint Lucia	1,032	13.7	55.6%				
		Egypt	4,476	13.2	48.5%				
		Saint Vincent and Grenadines	1,124	13.5	54.2%				
		Djibouti	928	14.3	40.5				
		Morocco	1916	14	47.9				
		Myanmar	2,212	13.6	50.5				
		Zambia	1,201	13.9	49.7				
		United Republic of Tanzania	1712	13	53.9				
		Venezuela	3,827	13.2	52.8				
		Grenada	1,244	13.7	57.9				
		Lebanon	4,415	13.6	53				
		China	8,313	13.7	49.6				
		Indonesia	2,979	13.8	50.7				
		Thailand	2,570	13.6	52.7				
		Uganda	1839	14.3	52.9				
		Tunisia	2,474	13.6	50.6				
		Botswana	1,336	14.3	54.4				
		Sri Lanka	2,435	13.7	50.5				
		India	7,120	13.9	42.5				
		Seychelles	1,095	13.6	50.9				
		Guyana	1,027	14.1	53.7				
		Ecuador	4,281	13.4	51.6				
		Jordan	1,542	14.4	54.5				
		Argentina	1,475	14.1	54.4				
		Kenya	2,694	13.9	53.4				
McMartin et al., 2012 [34]	Prospective cohort	Canada	3,757	10–11 years old	52%	Wave 1 (2003), Wave 2 (2006)	FV: FFQ over the past 12 months; number of daily servings of FV	Internalizing disorders that include common symptoms of depression and anxiety assessed by physician diagnosis	“none of the food items and nutrients including vegetable and fruit consumption … showed a statistically significant association with internalizing disorders.”
Park et al., 2018 [30]	Cross-sectional	Korea	65,528	14.99 (1.74)	48.4%	N/A	F, V: single-item dietary measure as part of the KYRBS how often students engaged in each dietary behaviour within the past 7 days	Depression symptoms assessed by a single question: “In the past 12 months, have you ever felt depression or hopelessness severe enough to compromise your daily activities during 2 weeks or more?”	“…healthier dietary behaviour [including frequent fruits (1 or more servings a day) and vegetables (3 or more times a day) consumption] was associated with … lower odds of perceived stress and depressive mood”
Winpenny et al., 2018 [32]	Prospective cohort (with longitudinal and cross-sectional analysis)	United Kingdom	603	14.05 (0.3)	60%	Wave 1 (2005–2007), Wave 2 (3 years later)	FV: 4 days diet diary, including two weekdays and two weekend days, reporting estimated portion sizes in terms of small, medium or large, household measures or as individual items	Depression assessed by the Moods and Feelings Questionnaire	“There were no significant associations between … fruit and vegetable intake … and depressive symptoms at baseline, nor … at 3-year follow up, after controlling for covariates”

a F, fruit intake; V, vegetable intake; FV, total fruit and vegetable intake.

b Youth Risk Behaviour Survey.

c ABAKQ, Adolescent Behaviours, Attitudes, and Knowledge Questionnaire.

d The study by Hoare et al. [28] also included prospective cohort data with the outcome of interest being adult depression. We omitted this part due to the nature of this systematic review. Moreover, there is an overlap between data used in [27] and data used for cross-sectional analysis in [29]. Both studies are included in this systematic review given that the sample size in [27] was 800 compared to 634 in [29].

e KYRBS, Korea Youth Risk Behaviour Web-based Survey.

f Saint Lucia, Egypt, Saint Vincent and Grenadines, Djibouti, Morocco, Myanmar, Zambia, United Republic of Tanzania, Venezuela, Grenada, Lebanon, China, Indonesia, Thailand, Uganda, Tunisia, Botswana, Sri Lanka, India, Seychelles, Guyana, Ecuador, Jordan, Argentina, Kenya.

g Global School-based Health Survey.

CES-D, Center for Epidemiologic Studies Depression Scale; COOPS/WONCA, Dartmouth COOP Functional Health Assessment charts/World Organization of Family Doctors; F, fruit intake, V, vegetables intake, FV, fruit and vegetables combined intake; N/A, not applicable; NR, not reported; SMFQ, Moods and Feelings Questionnaire.

Only self-reported dietary assessment instruments were used. Eight studies [23–25, 27–30, 35] assessed intakes of fruit and vegetables separately, while five studies [26, 29, 31, 32, 34] assessed combined FVI. Seven studies [23, 24, 27–30, 35] measured FVI in terms of frequency of FV consumption, while five studies [25, 26, 31, 32, 34] assessed FVI in terms of servings or grams per day. Food frequency questionnaire [34], four-day diet diary [32], and 24-h dietary recall [25] were used in one study each. Single-item dietary assessment questions were part of larger questionnaires on various lifestyle behaviours in nine studies [23, 24, 26–31, 35]. The questions referred to fruit, vegetable, or combined intake in the past 12 months [34, 35], 30 days [23, 29], 7 days [24, 28, 30], and the day before [26, 27, 31].

All studies assessed depression, two assessed anxiety [23, 29], and one study [34] included common symptoms of depression and anxiety (combined) as the outcome of interest. Six studies used the following validated questionnaires to assess the outcomes of interest: COOPS/WONCA questionnaire [25], SMFQ [26, 31], CES-D [27], Korean version of the Beck Depression Inventory [35], and Moods and Feelings Questionnaire [32]. One study [34] included physician diagnoses of internalizing disorders, and the rest of the studies [23, 24, 28–30] used single-item self-reported questions to assess the outcome(s) of interest. Subgroup analysis based on gender was conducted in five studies [25–27, 31, 32].

### Quality Assessment

Of 10 cross-sectional studies, eight [23–28, 31, 32] omitted the criteria for inclusion in the sample, and five [23, 24, 28–30] did not include validated scales to assess CMDs. Causal claims in respect to the focal association were made in two [23, 24] cross-sectional studies. All (except one [24]) studies identified and adjusted for at least some important confounding factors: four studies [25, 27, 28, 30] included indicators of SES and one [23] adjusted for food insecurity, two studies stratified by ethnicity [24] and gender [31].

There was one [35] case-control study. Community controls with no history of disease were selected from the same source population as the cases (i.e., adolescent youth attending the University Health Center for annual routine health examinations). FFQ with 63 food items and the Korean version of the Beck Depression Inventory (K-BDI) were included in the same questionnaire. Importantly, participants with pre-existing psychological conditions or those taking medication for depression were excluded from the study. Some of the most important confounding factors, such as SES, familial history of depression and physical activity, were not measured. It is unclear whether assessors were blinded to the research question or outcome assessment.

Among three prospective cohort studies, samples were representative of 10–11 years old children in corresponding communities in one study [34]. In all cohort studies, non-exposed cohorts were drawn from the same community as exposed cohorts and samples appeared free from mental disorders at the beginning of the studies. All studies ascertained exposure using self-reported dietary measures, and in all but one [34] mental disorders were self-reported. One study [31] included a two-year follow-up, while the other two studies included three-year [32, 34] follow-ups. One study [34] had complete follow up. Another study [31] had 74.5% participation rate, but participants lost to follow-up (3.2% refusal, 7.2% unavailable, 15.1% relocated) were unlikely to introduce bias. One study [32] reported a follow-up rate of less than 70%; the analysis of differences between participants and non-participants was not available, making it difficult to assess the possibility of selection bias. All studies adjusted for sex/gender. Other confounding factors adjusted for included: parental education, and the school participants attended [31]; age, SES, other lifestyle behaviours (smoking, physical activity, alcohol consumption, sleep), friendship quality, self-esteem, family functioning, %body fat, medication use, total energy intake, and depression symptoms at baseline [32]; and household income, parental marital status, parental education, body weight status, physical activity and geographic area [34].

### F&V Intakes (Measured Separately) and Depression

Eight studies (seven cross-sectional [23–25, 27–30] and one case-control [35]) reported inconsistent associations between fruit and vegetable intakes and depression symptoms. For example, the study by Liu et al. [29] that analyzed the association between depression and fruit and vegetable intakes in 25 low- and middle-income countries reported statistically significant associations. While the associations between fruit intake and depression were statistically significant for some countries (e.g., Tanzania, China, Indonesia, Thailand, India, Seychelles, Ecuador, and Jordan), they were not statistically significant for other low- and middle-income countries (Saint Lucia, Egypt, Saint Vincent and the Grenadines, Djibouti, Morocco, Myanmar, Zambia, Venezuela, Grenada, Lebanon, Uganda, Tunisia, Botswana, Sri Lanka, Guyana, Argentina, and Kenya). Associations in all listed countries were adjusted for different potential confounding factors. Liu et al. pooled data from all studies in a meta-analysis: fruit intake of <2 times and 2 or more times/day versus none was associated with 0.79 (0.73; 0.86) and 0.75 (0.68, 0.82) times lower odds of depression, respectively. Vegetable intake of <3 times/day and 3 or more times per day vs. none was associated with 0.74 (0.67; 0.83) and 0.75 (0.68; 0.84) times lower odds of depression, respectively.

### FVI (Combined) and Depression

Four studies reported on the association between combined FVI and depression. Two studies were cross-sectional, while two others included both cross-sectional and longitudinal analyses. One cross-sectional study [29] reported statistically significant associations in four of 25 low- and middle-income countries (i.e., Seychelles, Ecuador, Jordan, Kenya). One prospective study reported non-significant associations in univariate analyses: OR = 0.87 (95% CI 0.39; 1.96) for males and OR = 0.85 (95% CI 0.54; 1.34) for females [31]. Another prospective cohort study reported no statistically significant associations following adjustment for covariates: *ß* = 0.14 (95% CI of −0.15; 0.43) [32].

### F&V Intakes (Measured Separately) and Anxiety

Two studies [23, 29] examined the associations between fruit and vegetable intakes and anxiety. Arat [23] reported results for six of the low- and middle-income countries and found statistically significant associations in Botswana, Kenya (for fruit but not vegetable intake as the exposure of interest), Seychelles, Uganda, Tanzania, and Zambia. Another study by Liu et al. [29] used data from the same questionnaire as Arat [23], although for a narrower age range, reporting statistically significant associations between fruit intake and anxiety for Morocco, Tanzania, Venezuela China, Indonesia, Uganda, Tunisia, Sri Lanka, India, Ecuador, Jordan, Argentina, Kenya; and between vegetable intake and anxiety in Saint Vincent and the Grenadines, Djibouti, Lebanon, China, Seychelles, and Ecuador. When the measures of association were combined in a meta-analysis, the fruit intake of <2 times/day and 2 or more times a day compared to no intake was associated with 0.60 (0.54; 0.67) and 0.61 (0.54, 0.68) times lower odds of anxiety, while vegetable intake of <3 times/day and 3 or more times a day versus no intake was associated with 0.71 (0.63; 0.81) and 0.87 (0.73; 1.03) times lower odds of having anxiety symptoms. Neither of the studies reported on the association between the combined FVI and anxiety.

### FVI (Combined) and Depression and Anxiety (Combined)

One study [34] concluded that there was no statistically significant association between FVI and internalizing disorders when comparing second tertile to first tertile (IRR 1.04, 95% CI 0.71; 1.53) and third tertile to first tertile (IRR 1.25, 95% CI 0.8; 1.99). Analyses were adjusted for energy intake, gender, household income, parental marital status and education, body weight status, physical activity, and geographical area.

## Discussion

Fruit and vegetables have long been recognized for their beneficial effects on gastrointestinal health, weight management, prevention of cardiovascular and metabolic disorders, respiratory health, and high bone mineral density, among other conditions and diseases [36]. Moreover, FVI has recently been shown to be associated with lower risk of mental disorders in the general population [18, 37]. However, our systematic review did not confirm previous claims for the existing association between FVI and CMDs specifically in youth. Among 12 identified studies, one case-control and some of the cross-sectional studies pointed to significant associations between FVI and CMDs in youth, while none of the three prospective cohort studies showed significant associations after adjusting for confounding factors.

Previously proposed biological mechanisms to explain the association between FVI and CMDs revolve around the high content of fiber, nutrients (e.g., vitamin C), and phytochemicals (e.g., polyphenols, carotenoids) [36] found in vegetables and fruits, which are believed to have beneficial effects on neurotransmitter systems, neuronal plasticity [38], and gut health [39–41]. Although the aforementioned biological mechanisms appear plausible and are supported by studies in adults, the effects of FVI on CMDs may differ in youth due to the rapid brain development during adolescence [42]. Another potential explanation involves one of the existing theoretical models derived from life course epidemiology—i.e., the accumulation of risk model [43]. This model states that every additional year of exposure is associated with an increased risk of poor outcomes: this could explain why the association between FVI and CMDs becomes apparent later in life. Further research looking at the diet-mental health relationship through the lens of life course epidemiology is warranted. Methodological challenges, discussed below, could also explain our findings.

Consistent with other literature on the diet-mental health relationship [15, 18, 44], cross-sectional study design was the one most commonly used. While the cross-sectional study design can help generate hypotheses, in respect to the diet-mental health relationship this task has already been fulfilled. The inability to determine the temporal order of diet and mental disorders makes this study design of limited value to any etiological inferences [45]. Moreover, cross-sectional studies identified in this systematic review provided inconsistent conclusions, potentially due to adjusting for different confounding factors. As for the case-control study [35], the authors excluded those with pre-existing mental disorders, thus partly tackling the issue of reverse causality, but we cannot exclude the possibility of recall bias. Both exposure and outcome were assessed at the same time, and the study did not report whether those who completed the dietary assessment were blinded to participants’ outcomes or the research question itself. Given the aforementioned limitations inherent to cross-sectional and case-control study designs and in the absence of prevention trials (in part due to ethical and feasibility concerns), attention and efforts should be redirected to planning and conducting rigorous prospective cohort studies. We identified three prospective cohort studies and, given the incremental nature of research, more prospective cohort studies that address the methodological issues outlined below would be of value.

First of all, SES is an established confounder linked to both diet and mental disorders and therefore should be controlled for in all studies investigating this focal relationship; SES was measured and adjusted for in two [32, 34] out of three cohort studies included in this systematic review. At the same time, some of the variables (e.g., weight status indicators) that were treated as confounding factors could well be intermediate variables that we should not control for. Additionally, it is important to consider the nature of confounding factors (e.g., time-invariant such as ethnicity, race, sex vs. time-variant such as food security, parental mental health, family functioning), which could inform the choice of analytical methods (e.g., parametric G-formula) other than the standard regression models (e.g., linear and logistic regression models). Employing directed acyclic graphs [46] could help guide these pre-analysis steps and identify appropriate adjustment sets, minimize inappropriate adjustment, and invite external scrutiny to enhance the quality of work.

In addition, there are measurement errors associated with both self-reported diet and mental disorders. Given the potential for recall and social desirability biases associated with self-report measures, sensitivity analyses to delineate the effects of measurement errors on the focal relationship are needed [47]. Adjustment for total energy intake is another strategy that has been strongly recommended to partially correct for the measurement error associated with self-reported dietary intake. Moreover, fruit juice is excluded from recent healthy eating recommendations due to excess free sugars they contain [48]; for this reason, consumption of fruit juice should not count toward FVI. In addition, validated questionnaires, as opposed to single-item screener questions, should be preferred for the assessment of mental health disorders. Lastly, despite pronounced sex differences in both the prevalence of mental disorders [49] and eating behaviours and diet [50, 51], sub-group analysis was done in less than half of the included studies, and further exploration of potential effect modification is of value.

## Conclusion

This systematic review showed that while inconsistent associations between FVI and CMDs in youth were reported in cross-sectional and case-control studies, no association was detected in prospective cohort studies. This evidence differs from what has recently been concluded in a systematic review on the association between FVI and depression in adults [18], which can be explained by the accumulation of risk theoretical model of the development of mental disorders and/or methodological challenges outlined in the paper.
