# Supplementary material for: Relationship Between Fruit and Vegetables Intake and Common Mental Disorders in Youth: A Systematic Review
Source: Public Health Rev. 2022 Sep 20;43:1604686. doi: 10.3389/phrs.2022.1604686 (PMC9530034; doi:10.3389/phrs.2022.1604686)
Supplement: Supplementary file 1 [file DataSheet1.docx]

**Public Health Reviews**

**Title:** Relationship between fruit and vegetables intake and common mental disorders in youth: a systematic review

**Supplementary File 1**

These search strategies use two filters, which were adapted for each database:

Campbell S. A filter to retrieve studies related to fruits from the OVID MEDLINE Database. John W. Scott Health Sciences Library, University of Alberta. Rev. Oct 7, 2019. https://docs.google.com/document/d/1S2AtLS76jaJxK0Pi_UOThdbXZYQiaO1WKddIOj4hLpE/edit

Campbell S. A filter to retrieve studies related to vegetables from the OVID MEDLINE Database. John W. Scott Health Sciences Library, University of Alberta. Rev. Oct 10, 2019. https://docs.google.com/document/d/19vrxZwZ5QHp030Ic55WUm6thtMdvM_tjBs7FrG3EJXM/edit

**Ovid MEDLINE(R) ALL <1946 to October 30, 2020>**

| **#** | **Search Statement** | **Results** |
| --- | --- | --- |
| 1 | *Fruit/ | 21758 |
| 2 | ("high fiber" or "high fibre" or "dietary fiber" or "dietary fibre" or FRUIT or fruits or ACAI or ACKEE or "ACTINIDIA ARGUTA" or "AIELE FRUIT*" or AMLA or (APPLE* not (phone* or computer* or smartphone* or device*)) or APRICOTS or apricot or ARONIA or avocado* or BABACO or BACURI or "BAEL FRUIT" or BANANA* or BERRIES or Blueberries or barberr* or brambleberr* or BREADFRUIT or BUSH BUTTER or "CACTUS PEAR* ORCAJA" or "CAMU CAMU" or clementines or GOOSEBERRIES or CARAMBOLAS or "CASHEW APPLE*" or CHERIMOYA or CHERRIES or "CHERRY LAUREL" or CITRUS or ORANGES or CITRONS or nectarine* or TAMURANA or currants or Dragonfruit* or GALGALS or grapes or GRAPEFRUIT* or "KI MIKAN" or KUMQUAT* or LEMONS or lemons or LIMES or lingonberr* or ORTANIQUE or POMELOS or TANGELO or TANGOR or YUZU or COCONA or CUPUACU or "CURCULIGO LATIFOLIA ORDATES" or DURIAN or FEIJOA or FIGS or FORTUNELLA or GUAVAS or HAWTHORN or JABOTICABA or "JAK FRUITS" or "JELLY FIGS" or KIWANO or KIWIFRUIT or "kiwi fruit*" or LANGSAT or LITCHIS or LONGANS or LOQUATS or MAMEY or MANGOES or MANGOSTEENS or MARULA or MEDLARS or medlar or MELON or melons or CANTALOUPE* or HONEYDEW or MUSKMELONS or MYROBALANS or MYRTLE or NARANJILLA or NONI or OLIVES or OMIJA or PAPAYAS or "PASSION FRUIT*" or passionfruit* or PEACHES or peach or PEARS or PEPINO or PERSIMMON* or PHALSA or PINEAPPLE* or PITAYOS or PLUMS or plum or POMEGRANATE* or "PRICKLY PEAR*" or prune or prunes or QUINCE or quinces or RAMBUTAN or rhubarb or rockmelon* or raspberr* or ROSEHIP* or SAPODILLA* or SASKATOONS or "saskatoon berr*" or "SEA BUCKTHORN*" or SLOES or SOURSOP or SPONDIAS or STARfruit* or strawberr* or tangerine* or tangelo* or TAMARIND* or TOMATILLO* or TOMATO* or WATERMELON* or EGUSI or bearberr* or soapberr* or salmonberr* or crowberr* or huckleberr* or haskap or cranberr* or chokecherr* or pincherr* or cloudberr* or dewberr* or elderberr* or "partridge berr*").ti,ab. | 169251 |
| 3 | *Vegetables/ | 12846 |
| 4 | vegetable*.ti,ab. | 53185 |
| 5 | (Artichoke* or arugula or Asparagus or aubergine* or "bamboo shoot*" or basil or bean or beans or beet or beets or beetroot* or "bok choy" or broadbean* or broccoflower or broccoli or "brussel* sprout*" or cabbage* or caraway or carrot* or cauliflower* or capsicum* or celeriac or celery* or chickpea* or chives or cilantro or collard* or coriander or corn or courgette* or cucumber* or daikon or edemame or eggplant* or endive or endives or fennel or fiddlehead* or garbanzos or garlic or ginger or greens or jimcama or kale or kohlrabi or leek* or legume* or lentil or lentils or lettuce* or okra or onion* or oregano or peas or parsley or parsnip* or pepper* or potato* or pumpkin* or radish* or radicchio or rutabaga* or salad or salads or salsify or scallion* or shallot* or sorrel or soybean* or spinach or squash or sweetcorn or "Swiss chard" or taro or tomatillo or topinambur or turnip* or "water chestnut*" or watercress or yam or yams or zucchini*).ti,ab. | 197952 |
| 6 | 1or 2 or 3 or 4 o4 5 | 367920 |
| 7 | Anxiety/ or *Mental Health/ | 98344 |
| 8 | hypervigilance.ti,ab. | 676 |
| 9 | anxiety disorders/ or agoraphobia/ or anxiety, separation/ or neurocirculatory asthenia/ or neurotic disorders/ or obsessive-compulsive disorder/ or hoarding disorder/ or panic disorder/ or phobic disorders/ or phobia, social/ | 77949 |
| 10 | anxiety.ti,ab. | 180274 |
| 11 | agoraphobia.ti,ab. | 3012 |
| 12 | neurocirculatory asthenia.ti,ab. | 264 |
| 13 | neurotic disorder*.ti,ab. | 806 |
| 14 | Depression/ | 114471 |
| 15 | depressive disorder/ or depressive disorder, major/ or dysthymic disorder/ or seasonal affective disorder/ | 101154 |
| 16 | depressive.ti,ab. | 111053 |
| 17 | depression.ti,ab. | 319066 |
| 18 | dysthymic disorder*.ti,ab. | 766 |
| 19 | ("seasonal affective disorder*" or "internali?ing symptoms" or "common mental disorders" or cmd or cmds).ti,ab. | 6855 |
| 20 | 7 or 8 or 9 or 10 or 11 or 12 or 13 or 14 or 15 or 16 or 17 or 18 or 19 | 574885 |
| 21 | 6 and 20 | 2362 |
| 22 | limit 21 to ("child (6 to 12 years)" or "adolescent (13 to 18 years)") | 258 |
| 23 | limit 21 to ("newborn infant (birth to 1 month)" or "infant (1 to 23 months)" or "preschool child (2 to 5 years)" or "all adult (19 plus years)") | 720 |
| 24 | 22 and 23 | 175 |
| 25 | (21 not 23) or 24 | 1817 |
| 26 | (youth or youths or adolescen* or teen* or high school* or middle school* or tween or tweens or young people or pubescent or pubescence or pre-pubescen*).mp. [mp=title, abstract, original title, name of substance word, subject heading word, floating sub-heading word, keyword heading word, organism supplementary concept word, protocol supplementary concept word, rare disease supplementary concept word, unique identifier, synonyms] | 2109818 |
| 27 | 21 and 26 | 255 |
| 28 | 25 or 27 | 1825 |
| 29 | exp Laboratory Animals/ or exp Disease Models, Animal/ or (rat or rats or mouse or mice or rabbit* or cat or cats or dog or dogs or pig or pigs or piglet* or procine or canine or rodent* or feline* or sheep or ewe or lamb or goat or goats or cow or cows or cattle or bovine or horse or horses or equine or fish or fishes or "Afar Depression" or "Great Depression" or "inbreeding depression" or "orange fluorescence" or "MdPI depression" or "respiratory depression" or "fruit bat*" or duck or ducks or bird or birds or turkey* or chicken* or insect* or "fruit fly" or "mito* depression" or aromatherapy or "Agent Orange" or "Orange County" or Cherry Valley or "Medlars Service").mp. | 5431331 |
| 30 | 28 not 29 | 792 |
| 31 | remove duplicates from 30 | 788 |

**Embase OVID<1974 to 2020 October 30>**

| **#** | **Search Statement** | **Results** |
| --- | --- | --- |
| 1 | exp *Fruit/ or "fruit vegetable"/ | 45785 |
| 2 | ("high fiber" or "high fibre" or "dietary fiber" or "dietary fibre" or FRUIT or fruits or ACAI or ACKEE or "ACTINIDIA ARGUTA" or "AIELE FRUIT*" or AMLA or (APPLE* not (phone* or computer* or smartphone* or device*)) or APRICOTS or apricot or ARONIA or avocado* or BABACO or BACURI or "BAEL FRUIT" or BANANA* or BERRIES or Blueberries or barberr* or brambleberr* or BREADFRUIT or BUSH BUTTER or "CACTUS PEAR*" or CAJA or "CAMU CAMU" or clementines or GOOSEBERRIES or CARAMBOLAS or "CASHEW APPLE*" or CHERIMOYA or CHERRIES or "CHERRY LAUREL" or CITRUS or ORANGES or CITRONS or nectarine* or TAMURANA or currants or Dragonfruit* or GALGALS or grapes or GRAPEFRUIT* or "KI MIKAN" or KUMQUAT* or LEMONS or lemons or LIMES or lingonberr* or ORTANIQUE or POMELOS or TANGELO or TANGOR or YUZU or COCONA or CUPUACU or "CURCULIGO LATIFOLIA" or DURIAN or FEIJOA or FORTUNELLA or GUAVAS or HAWTHORN or JABOTICABA or "JAK FRUITS" or "JELLY FIGS" or KIWANO or KIWIFRUIT or "kiwi fruit*" or LANGSAT or LITCHIS or LONGANS or LOQUATS or MAMEY or MANGOES or MANGOSTEENS or MARULA or MEDLARS or medlar or MELON or melons or CANTALOUPE* or HONEYDEW or MUSKMELONS or MYROBALANS or MYRTLE or NARANJILLA or NONI or OLIVES or OMIJA or PAPAYAS or "PASSION FRUIT*" or passionfruit* or PEACHES or peach or PEARS or PEPINO or PERSIMMON* or PHALSA or PINEAPPLE* or PITAYOS or PLUMS or plum or POMEGRANATE* or "PRICKLY PEAR*" or prune or prunes or QUINCE or quinces or RAMBUTAN or rhubarb or rockmelon* or raspberr* or ROSEHIP* or SAPODILLA* or SASKATOONS or "saskatoon berr*" or "SEA BUCKTHORN*" or SLOES or SOURSOP or SPONDIAS or STARfruit* or strawberr* or tangerine* or tangelo* or TAMARIND* or TOMATILLO* or TOMATO* or WATERMELON* or EGUSI or bearberr* or soapberr* or salmonberr* or crowberr* or huckleberr* or haskap or cranberr* or chokecherr* or pincherr* or cloudberr* or dewberr* or elderberr* or "partridge berr*").ti,ab. | 193922 |
| 3 | exp *Vegetable/ | 73915 |
| 4 | vegetable*.ti,ab. | 66674 |
| 5 | (Artichoke* or arugula or Asparagus or aubergine* or "bamboo shoot*" or basil or bean or beans or beet or beets or beetroot* or "bok choy" or broadbean* or broccoflower or broccoli or "brussel* sprout*" or cabbage* or caraway or carrot* or cauliflower* or capsicum* or celeriac or celery* or chickpea* or chives or cilantro or collard* or coriander or corn or courgette* or cucumber* or daikon or edemame or eggplant* or endive or endives or fennel or fiddlehead* or garbanzos or garlic or ginger or greens or jimcama or kale or kohlrabi or leek* or legume* or lentil or lentils or lettuce* or okra or onion* or oregano or peas or parsley or parsnip* or pepper* or potato* or pumpkin* or radish* or radicchio or rutabaga* or sage or salad or salads or salsify or scallion* or shallot* or sorrel or soybean* or spinach or squash or sweetcorn or "Swiss chard" or taro or tomatillo or topinambur or turnip* or "water chestnut*" or watercress or yam or yams or zucchini*).ti,ab. | 222984 |
| 6 | 1 or 2 or 3 or 4 or 5 | 444131 |
| 7 | anxiety/ or mental health/ or psychological well-being/ | 328780 |
| 8 | hypervigilance.ti,ab. | 960 |
| 9 | anxiety disorder/ or generalized anxiety disorder/ or "mixed anxiety and depression"/ | 75307 |
| 10 | anxiety.ti,ab. | 259621 |
| 11 | agoraphobia.ti,ab. | 3984 |
| 12 | neurocirculatory [asthenia.mp](http://asthenia.mp/). | 162 |
| 13 | neurotic disorder*.mp. | 1224 |
| 14 | depression/ or adolescent depression/ or atypical depression/ or chronic depression/ or dysphoria/ or dysthymia/ or major depression/ or minor depression/ or "mixed anxiety and depression"/ or seasonal affective disorder/ | 411982 |
| 15 | depressive disorder*.ti,ab. | 47762 |
| 16 | dysthymic disorder*.ti,ab. | 969 |
| 17 | ("seasonal affective disorder*" or "common mental disorders" or cmd or cmds).ti,ab. | 6652 |
| 18 | 7 or 8 or 9 or 10 or 11 or 12 or 13 or 14 or 15 or 16 or 17 | 745785 |
| 19 | 6 and 18 | 4290 |
| 20 | (youth or youths or adolescen* or teen* or high school* or middle school* or tween or tweens or young people or pubescent or pubescence or pre-pubescen*).mp. [mp=title, abstract, heading word, drug trade name, original title, device manufacturer, drug manufacturer, device trade name, keyword, floating subheading word, candidate term word] | 1659610 |
| 21 | limit 19 to (adult <18 to 64 years> or aged <65+ years>) | 2080 |
| 22 | 19 | 4290 |
| 23 | limit 19 to (embryo <first trimester> or infant <to one year> or child <unspecified age> or preschool child <1 to 6 years>) | 387 |
| 24 | 21 or 23 | 2357 |
| 25 | limit 24 to (school child <7 to 12 years> or adolescent <13 to 17 years>) | 372 |
| 26 | 19 not 24 | 1933 |
| 27 | 25 or 26 | 2305 |
| 28 | 19 and 20 | 621 |
| 29 | 27 or 28 | 2366 |
| 30 | exp animal experiment/ or exp animal model/ or exp animal disease/ or (animal* or rat or rats or mouse or mice or rabbit* or cat or cats or dog or dogs or pig or pigs or piglet* or procine or canine or rodent* or feline* or sheep or ewe or lamb or goat or goats or cow or cows or cattle or bison* or buffalo* bovine or horse or horses or equine or fish or fishes or "Afar Depression" or "Great Depression" or "inbreeding depression" or "orange fluorescence" or "MdPI depression" or "fruit bat*" or duck or ducks or bird or birds or turkey* or chicken* or insect* or "fruit fly" or "mito* depression" or aromatherapy or "Agent Orange" or "Orange County" or Cherry Valley or "Medlars Service" or dementia* or alzheimer* or "sage publication*").mp. | 7253684 |
| 31 | 29 not 30 | 968 |
| 32 | remove duplicates from 31 | 960 |

|  |
| --- |
|  |

**PsycINFO OVID <1806 to October 30 2020>**

| # | Search Statement | **Results** |
| --- | --- | --- |
| 1 | (fruit or fruits).ti,ab. [mp=title, abstract, heading word, table of contents, key concepts, original title, tests & measures, mesh] | 8832 |
| 2 | ("high fiber" or "high fibre" or "dietary fiber" or "dietary fibre" or FRUIT or fruits or ACAI or ACKEE or "ACTINIDIA ARGUTA" or "AIELE FRUIT*" or AMLA or (APPLE* not (phone* or computer* or smartphone* or device*)) or APRICOTS or apricot or ARONIA or avocado* or BABACO or BACURI or "BAEL FRUIT" or BANANA* or BERRIES or Blueberries or barberr* or brambleberr* or BREADFRUIT or BUSH BUTTER or "CACTUS PEAR*" or CAJA or "CAMU CAMU" or clementines or GOOSEBERRIES or CARAMBOLAS or "CASHEW APPLE*" or CHERIMOYA or CHERRIES or "CHERRY LAUREL" or CITRUS or ORANGES or CITRONS or nectarine* or TAMURANA or currants or Dragonfruit* or GALGALS or grapes or GRAPEFRUIT* or "KI MIKAN" or KUMQUAT* or LEMONS or lemons or LIMES or lingonberr* or ORTANIQUE or POMELOS or TANGELO or TANGOR or YUZU or COCONA or CUPUACU or "CURCULIGO LATIFOLIA" or DURIAN or FEIJOA or FORTUNELLA or GUAVAS or HAWTHORN or JABOTICABA or "JAK FRUITS" or "JELLY FIGS" or KIWANO or KIWIFRUIT or "kiwi fruit*" or LANGSAT or LITCHIS or LONGANS or LOQUATS or MAMEY or MANGOES or MANGOSTEENS or MARULA or MEDLARS or medlar or MELON or melons or CANTALOUPE* or HONEYDEW or MUSKMELONS or MYROBALANS or MYRTLE or NARANJILLA or NONI or OLIVES or OMIJA or PAPAYAS or "PASSION FRUIT*" or passionfruit* or PEACHES or peach or PEARS or PEPINO or PERSIMMON* or PHALSA or PINEAPPLE* or PITAYOS or PLUMS or plum or POMEGRANATE* or "PRICKLY PEAR*" or prune or prunes or QUINCE or quinces or RAMBUTAN or rhubarb or rockmelon* or raspberr* or ROSEHIP* or SAPODILLA* or SASKATOONS or "saskatoon berr*" or "SEA BUCKTHORN*" or SLOES or SOURSOP or SPONDIAS or STARfruit* or strawberr* or tangerine* or tangelo* or TAMARIND* or TOMATILLO* or TOMATO* or WATERMELON* or EGUSI or bearberr* or soapberr* or salmonberr* or crowberr* or huckleberr* or haskap or cranberr* or chokecherr* or pincherr* or cloudberr* or dewberr* or elderberr* or "partridge berr*").ti,ab. | 13299 |
| 3 | vegetable*.ti,ab. | 5753 |
| 4 | (Artichoke* or arugula or Asparagus or aubergine* or "bamboo shoot*" or basil or bean or beans or beet or beets or beetroot* or "bok choy" or broadbean* or broccoflower or broccoli or "brussel* sprout*" or cabbage* or caraway or carrot* or cauliflower* or capsicum* or celeriac or celery* or chickpea* or chives or cilantro or collard* or coriander or corn or courgette* or cucumber* or daikon or edemame or eggplant* or endive or endives or fennel or fiddlehead* or garbanzos or garlic or ginger or greens or jimcama or kale or kohlrabi or leek* or legume* or lentil or lentils or lettuce* or okra or onion* or oregano or peas or parsley or parsnip* or pepper* or potato* or pumpkin* or radish* or radicchio or rutabaga* or salad or salads or salsify or scallion* or shallot* or sorrel or soybean* or spinach or squash or sweetcorn or "Swiss chard" or taro or tomatillo or topinambur or turnip* or "water chestnut*" or watercress or yam or yams or zucchini*).ti,ab. | 5852 |
| 5 | 1 or 2 or 3 or 4 | 19567 |
| 6 | Anxiety/ | 59895 |
| 7 | [hypervigilance.mp](http://hypervigilance.mp/). | 856 |
| 8 | anxiety disorders/ or generalized anxiety disorder/ or internalizing symptoms/ | 21251 |
| 9 | [anxiety.mp](http://anxiety.mp/). | 228158 |
| 10 | [agoraphobia.mp](http://agoraphobia.mp/). | 5659 |
| 11 | neurocirculatory [asthenia.mp](http://asthenia.mp/). | 135 |
| 12 | neurotic disorder*.mp. | 7327 |
| 13 | "depression (emotion)"/ | 25292 |
| 14 | major depression/ or dysthymic disorder/ or atypical depression/ or internalizing symptoms/ or seasonal affective disorder/ or mental health/ or primary mental health prevention/ | 182269 |
| 15 | [depressive.mp](http://depressive.mp/). | 106924 |
| 16 | (depression or ("common mental disorders" or cmd or cmds)).mp. | 321309 |
| 17 | dysthymic disorder*.mp. | 2517 |
| 18 | "seasonal affective disorder*".mp. | 1579 |
| 19 | 6 or 7 or 8 or 9 or 10 or 11 or 12 or 13 or 14 or 15 or 16 or 17 or 18 | 520128 |
| 20 | 5 and 19 | 988 |
| 21 | limit 20 to (120 neonatal <birth to age 1 mo> or 140 infancy <2 to 23 mo> or 160 preschool age <age 2 to 5 yrs>) | 23 |
| 22 | 20 | 988 |
| 23 | limit 22 to "300 adulthood <age 18 yrs and older>" | 490 |
| 24 | 21 or 23 | 502 |
| 25 | limit 24 to (180 school age <age 6 to 12 yrs> or 200 adolescence <age 13 to 17 yrs>) | 49 |
| 26 | (20 not 24) or 25 | 535 |
| 27 | 20 and (youth or youths or adolescen* or teen* or high school* or middle school* or tween or tweens or young people or pubescent or pubescence or pre-pubescen*).mp. | 164 |
| 28 | 26 or 27 | 589 |
| 29 | exp Animal Models/ or (animal* or rat or rats or mouse or mice or rabbit* or cat or cats or dog or dogs or pig or pigs or piglet* or procine or canine or rodent* or feline* or sheep or ewe or lamb or goat or goats or cow or cows or cattle or bison* or buffalo* bovine or horse or horses or equine or fish or fishes or "Afar Depression" or "Great Depression" or "inbreeding depression" or "orange fluorescence" or "MdPI depression" or "fruit bat*" or duck or ducks or bird or birds or turkey* or chicken* or insect* or "fruit fly" or "mito* depression" or aromatherapy or "Agent Orange" or "Orange County" or Cherry Valley or "Medlars Service" or dementia* or alzheimer*).mp. or ("older adult*" or elderly).ti. | 609390 |
| 30 | 28 not 29 | 419 |
| 31 | remove duplicates from 30 | 419 |

**FSTA (WOS) Timespan=All Searched October 30, 2020**

| S1 | DESCRIPTORS: (FRUITS OR ACAI OR ACKEE OR ACTINIDIA ARGUTA OR AIELE FRUITS OR AMLA OR APPLES OR APRICOTS OR ARONIA OR BABACO OR BACURI OR BAEL FRUIT OR BANANAS OR BARBADOS CHERRIES OR BERRIES OR BREADFRUIT OR BUSH BUTTER OR CACTI OR CACTUS PEARS OR CAJA OR CAMU-CAMU OR CAPE GOOSEBERRIES OR CARAMBOLAS OR CASHEW APPLES OR CHERIMOYA OR CHERRIES OR CHERRY LAUREL OR CITRUS FRUITS OR COCONA OR CORNELIAN CHERRIES OR CUPUACU OR CURCULIGO LATIFOLIA OR CUSTARD APPLES OR DATES OR DURIAN OR EXOTIC FRUITS OR FEIJOA OR FIGS OR FORTUNELLA OR GUAVAS OR HAWTHORN FRUITS OR JABOTICABA OR JAK FRUITS OR JAPANESE APRICOTS OR JELLY FIGS OR JUJUBES OR KIWANO OR KIWIFRUIT OR LANGSAT OR LITCHIS OR LONGANS OR LOQUATS OR MALAY APPLES OR MAMEY OR MANGOES OR MANGOSTEENS OR MARULA OR MEDLARS OR MELONS OR MYROBALANS OR MYRTLE OR NARANJILLA OR NONI OR OLIVES OR OMIJA OR PAPAYAS OR PASSION FRUITS OR PEACHES OR PEARS OR PEPINO OR PERSIMMONS OR PHALSA OR PINEAPPLES OR PITAYOS OR PLUMS OR POME FRUITS OR POMEGRANATES OR PRICKLY PEARS OR QUINCES OR RAMBUTAN OR ROSE APPLES OR ROSEHIPS OR SAPODILLAS OR SASKATOON FRUITS OR SEA BUCKTHORN OR SLOES OR SOURSOP OR SPONDIAS OR STAR APPLES OR STONE FRUITS OR SUGAR APPLES OR TAMARILLOS OR TAMARINDS OR TOMATILLOS OR TOMATOES OR TROPICAL FRUITS OR WATERMELONS OR WILD FRUITS) OR TOPIC: (("high fiber" or "high fibre" or "dietary fiber" or "dietary fibre" or FRUIT or fruits or ACAI or ACKEE or "ACTINIDIA ARGUTA" or "AIELE FRUIT*" or AMLA or (APPLE* not (phone* or computer* or smartphone* or device*)) or APRICOTS or apricot or ARONIA or avocado* or BABACO or BACURI or "BAEL FRUIT" or BANANA* or BERRIES or Blueberries or barberr* or brambleberr* or BREADFRUIT or BUSH BUTTER or "CACTUS PEAR*" or CAJA or "CAMU CAMU" or clementines or GOOSEBERRIES or CARAMBOLAS or "CASHEW APPLE*" or CHERIMOYA or CHERRIES or "CHERRY LAUREL" or CITRUS or ORANGES or CITRONS or nectarine* or TAMURANA or currants or Dragonfruit* or GALGALS or grapes or GRAPEFRUIT* or "KI MIKAN" or KUMQUAT* or LEMONS or lemons or LIMES or lingonberr* or ORTANIQUE or POMELOS or TANGELO or TANGOR or YUZU or COCONA or CUPUACU or "CURCULIGO LATIFOLIA" or DURIAN or FEIJOA or FORTUNELLA or GUAVAS or HAWTHORN or JABOTICABA or "JAK FRUITS" or "JELLY FIGS" or KIWANO or KIWIFRUIT or "kiwi fruit*" or LANGSAT or LITCHIS or LONGANS or LOQUATS or MAMEY or MANGOES or MANGOSTEENS or MARULA or MEDLARS or medlar or MELON or melons or CANTALOUPE* or HONEYDEW or MUSKMELONS or MYROBALANS or MYRTLE or NARANJILLA or NONI or OLIVES or OMIJA or PAPAYAS or "PASSION FRUIT*" or passionfruit* or PEACHES or peach or PEARS or PEPINO or PERSIMMON* or PHALSA or PINEAPPLE* or PITAYOS or PLUMS or plum or POMEGRANATE* or "PRICKLY PEAR*" or prune or prunes or QUINCE or quinces or RAMBUTAN or rhubarb or rockmelon* or raspberr* or ROSEHIP* or SAPODILLA* or SASKATOONS or "saskatoon berr*" or "SEA BUCKTHORN*" or SLOES or SOURSOP or SPONDIAS or STARfruit* or strawberr* or tangerine* or tangelo* or TAMARIND* or TOMATILLO* or TOMATO* or WATERMELON* or EGUSI or bearberr* or soapberr* or salmonberr* or crowberr* or huckleberr* or haskap or cranberr* or chokecherr* or pincherr* or cloudberr* or dewberr* or elderberr* or "partridge berr*")) OR TOPIC: ((Artichoke* or arugula or Asparagus or aubergine* or "bamboo shoot*" or basil or bean or beans or beet or beets or beetroot* or "bok choy" or broadbean* or broccoflower or broccoli or "brussel* sprout*" or cabbage* or caraway or carrot* or cauliflower* or capsicum* or celeriac or celery* or chickpea* or chives or cilantro or collard* or coriander or corn or courgette* or cucumber* or daikon or edemame or eggplant* or endive or endives or fennel or fiddlehead* or garbanzos or garlic or ginger or greens or jimcama or kale or kohlrabi or leek* or legume* or lentil or lentils or lettuce* or okra or onion* or oregano or peas or parsley or parsnip* or pepper* or potato* or pumpkin* or radish* or radicchio or rutabaga* or salad or salads or salsify or scallion* or shallot* or sorrel or soybean* or spinach or squash or sweetcorn or "Swiss chard" or taro or tomatillo or topinambur or turnip* or "water chestnut*" or watercress or yam or yams or zucchini* or vegetable*)) OR DESCRIPTORS: (VEGETABLES OR DRIED PEAS OR DRIED VEGETABLES OR VEGETABLES SPECIFIC OR ALLIUM OR AMARANTH LEAVES OR ANU OR ARRACACHA OR ARROWHEAD OR ARTICHOKES OR ASPARAGUS OR AUBERGINES OR AVOCADOS OR BAMBOO OR BAOBAB OR BEETS OR BITTER LEAF OR BLACK SALSIFY OR BORAGE OR BOXTHORN OR BRASSICA OR BROCCOLI OR BRUSSELS SPROUTS OR BURDOCK OR BUTTERBUR OR CABBAGES OR CANNA OR CAPSICUMS OR CARDOONS OR CARROTS OR CASSAVA OR CAULIFLOWERS OR CELERIAC OR CELERY OR CEYLON SPINACH OR CHAYOTE OR CHICORY (VEGETABLES) OR CHINESE CABBAGES OR CHINESE CHIVES OR CHINESE WATER CHESTNUTS OR CHUFA NUTS OR COCOYAMS OR CORCHORUS OLITORIUS OR COURGETTES OR CRESS OR CRUCIFERAE OR CUCUMBERS OR CUCURBITACEAE OR CURCUMA OR DANDELIONS OR EDIBLE FLOWERS OR ELEPHANT YAMS OR ENDIVES OR ERUCA SATIVA OR EWEDU OR EXOTIC VEGETABLES OR FENNEL OR FENUGREEK OR FUNGI EDIBLE OR GHERKINS OR GLOBE ARTICHOKES OR GODULBAEGI OR GOURDS OR GREEN VEGETABLES OR HOUTTUYNIA CORDATA OR INDIAN SPINACH OR JERUSALEM ARTICHOKES OR KALE OR KOHLRABI OR KOMATSUNA OR KUDZU OR LEAFY VEGETABLES OR LEEKS OR LEGUMES OR LETTUCES OR LILY BULBS OR LOTUS ROOTS OR MACA OR MARROWS OR MUSTARD GREENS OR NETTLES OR OCA OR OKRA OR ONIONS OR PALM HEARTS OR PARSNIPS OR PERILLA OR POTATOES OR PUMPKINS OR PURSLANE OR RADISHES OR RAKKYO OR RHUBARB OR ROCKET OR ROOT CROPS OR ROOT VEGETABLES OR SALAD VEGETABLES OR SALSIFY OR SCALLION OR SEA KALE OR SESBANIA OR SHALLOTS OR SOLANUM OR SPINACH OR SPROUTS OR SQUASHES OR SWEDES OR SWEET POTATOES OR SWEETCORN OR SWISS CHARD OR TUBERS OR TURNIPS OR ULLUCO OR VINE LEAVES OR WATER CHESTNUTS OR WATER DROPWORT OR WATER SPINACH OR WATERCRESS OR WELSH ONIONS OR WILD CABBAGE OR WILD VEGETABLES OR YACON OR YAMS OR ZEDOARY) | 466,913 |
| --- | --- | --- |
| S2 | DESCRIPTORS: (DEPRESSION) OR TOPIC: ("neurotic disorder" or hypervigilance or agoraphobia or "dysthymic disorder" or anxiety or "common mental disorders" or cmd or "internalizing symptoms" or "internalising symptoms") OR TOPIC: ("mental illness" or "clinical depression" or "major depression" or "mental health") | 3,226 |
| S3 | #1 and #2 | 546 |
| S4 | DESCRIPTORS: (ANIMAL MODELS OR ANIMAL DISEASES) | 43,239 |
| S5 | TS=(animal* or rat or rats or mouse or mice or rabbit* or cat or cats or dog or dogs or pig or pigs or piglet* or procine or canine or rodent* or feline* or sheep or ewe or lamb or goat or goats or cow or cows or cattle or bison* or buffalo* bovine or horse or horses or equine or fish or fishes or"Afar Depression" or "Great Depression" or "inbreeding depression" or "orange fluorescence" or "MdPI depression" or "fruit bat*" or duck or ducks or bird or birds or turkey* or chicken* or insect* or "fruit fly" or "mito* depression" or aromatherapy or "Agent Orange" or "Orange County" or Cherry Valley or "Medlars Service" or dementia* or alzheimer*) | 304,628 |
| S6 | TI=("older adult*" or elderly) | 3,000 |
| S7 | #6 OR #5 OR #4 | 307,186 |
| S8 | #3 NOT #7 | 325 |

**Proquest Dissertations and These Global up to October 30, 2020**

| S1 | ti(("neurotic disorder" OR hypervigilance OR agoraphobia OR "dysthymic disorder" OR anxiety OR "common mental disorders" OR "internalizing symptoms" OR "internalising symptoms" OR "mental illness" OR "clinical depression" OR "major depression" OR "mental health")) | [22,](https://search-proquest-com.login.ezproxy.library.ualberta.ca/recentsearches.recentsearchtabview.recentsearchesgridview.scrolledrecentsearchlist.checkdbssearchlink_0:rerunsearch/D20CA5CA7E754A96PQ/None?site=pqdtglobal&t:ac=RecentSearches)904 |
| --- | --- | --- |
| S2 | [ti((("high fiber" OR "high fibre" OR "dietary fiber" OR "dietary fibre" OR FRUIT OR fruits OR ACAI OR ACKEE OR "ACTINIDIA ARGUTA" OR "AIELE FRUIT*" OR AMLA OR (APPLE* NOT (phone* OR computer* OR smartphone* OR device*)) OR APRICOTS OR apricot OR ARONIA OR avocado* OR BABACO OR BACURI OR "BAEL FRUIT" OR BANANA* OR BERRIES OR Blueberries OR barberr* OR brambleberr* OR BREADFRUIT OR BUSH BUTTER OR "CACTUS PEAR*" OR CAJA OR "CAMU CAMU" OR clementines OR GOOSEBERRIES OR CARAMBOLAS OR "CASHEW APPLE*" OR CHERIMOYA OR CHERRIES OR "CHERRY LAUREL" OR CITRUS OR ORANGES OR CITRONS OR nectarine* OR TAMURANA OR currants OR Dragonfruit* OR GALGALS OR grapes OR GRAPEFRUIT* OR "KI MIKAN" OR KUMQUAT* OR LEMONS OR lemons OR LIMES OR lingonberr* OR ORTANIQUE OR POMELOS OR TANGELO OR TANGOR OR YUZU OR COCONA OR CUPUACU OR "CURCULIGO LATIFOLIA" OR DURIAN OR FEIJOA OR FORTUNELLA OR GUAVAS OR HAWTHORN OR JABOTICABA OR "JAK FRUITS" OR "JELLY FIGS" OR KIWANO OR KIWIFRUIT OR "kiwi fruit*" OR LANGSAT OR LITCHIS OR LONGANS OR LOQUATS OR MAMEY OR MANGOES OR MANGOSTEENS OR MARULA OR MEDLARS OR medlar OR MELON OR melons OR CANTALOUPE* OR HONEYDEW OR MUSKMELONS OR MYROBALANS OR MYRTLE OR NARANJILLA OR NONI OR OLIVES OR OMIJA OR PAPAYAS OR "PASSION FRUIT*" OR passionfruit* OR PEACHES OR peach OR PEARS OR PEPINO OR PERSIMMON* OR PHALSA OR PINEAPPLE* OR PITAYOS OR PLUMS OR plum OR POMEGRANATE* OR "PRICKLY PEAR*" OR prune OR prunes OR QUINCE OR quinces OR RAMBUTAN OR rhubarb OR rockmelon* OR raspberr* OR ROSEHIP* OR SAPODILLA* OR SASKATOONS OR "saskatoon berries" OR "SEA BUCKTHORN*" OR SLOES OR SOURSOP OR SPONDIAS OR STARfruit* OR strawberr* OR tangerine* OR tangelo* OR TAMARIND* OR TOMATILLO* OR TOMATO* OR WATERMELON* OR EGUSI OR bearberr* OR soapberr* OR salmonberr* OR crowberr* OR huckleberr* OR haskap OR cranberr* OR chokecherr* OR pincherr* OR cloudberr* OR dewberr* OR elderberr* OR "partride berries" OR artichoke* OR arugula OR Asparagus OR aubergine* OR "bamboo shoot*" OR basil OR bean OR beans OR beet OR beets OR beetroot* OR "bok choy" OR broadbean* OR broccoflower OR broccoli OR "brussel* sprout*" OR cabbage* OR caraway OR carrot* OR cauliflower* OR capsicum* OR celeriac OR celery* OR chickpea* OR chives OR cilantro OR collard* OR coriander OR corn OR courgette* OR cucumber* OR daikon OR edemame OR eggplant* OR endive OR endives OR fennel OR fiddlehead* OR garbanzos OR garlic OR ginger OR jimcama OR kale OR kohlrabi OR leek* OR legume* OR lentil OR lentils OR lettuce* OR okra OR onion* OR oregano OR peas OR parsley OR parsnip* OR pepper* OR potato* OR pumpkin* OR radish* OR radicchio OR rutabaga* OR salad OR salads OR salsify OR scallion* OR shallot* OR sorrel OR soybean* OR spinach OR squash OR sweetcorn OR "Swiss chard" OR taro OR tomatillo OR topinambur OR turnip* OR "water chestnut*" OR watercress OR yam OR yams OR zucchini* OR vegetable*))) OR ab((("high fiber" OR "high fibre" OR "dietary fiber" OR "dietary fibre" OR FRUIT OR fruits OR ACAI OR ACKEE OR "ACTINIDIA ARGUTA" OR "AIELE FRUIT*" OR AMLA OR (APPLE* NOT (phone* OR computer* OR smartphone* OR device*)) OR APRICOTS OR apricot OR ARONIA OR avocado* OR BABACO OR BACURI OR "BAEL FRUIT" OR BANANA* OR BERRIES OR Blueberries OR barberr* OR brambleberr* OR BREADFRUIT OR BUSH BUTTER OR "CACTUS PEAR*" OR CAJA OR "CAMU CAMU" OR clementines OR GOOSEBERRIES OR CARAMBOLAS OR "CASHEW APPLE*" OR CHERIMOYA OR CHERRIES OR "CHERRY LAUREL" OR CITRUS OR ORANGES OR CITRONS OR nectarine* OR TAMURANA OR currants OR Dragonfruit* OR GALGALS OR grapes OR GRAPEFRUIT* OR "KI MIKAN" OR KUMQUAT* OR LEMONS OR lemons OR LIMES OR lingonberr* OR ORTANIQUE OR POMELOS OR TANGELO OR TANGOR OR YUZU OR COCONA OR CUPUACU OR "CURCULIGO LATIFOLIA" OR DURIAN OR FEIJOA OR FORTUNELLA OR GUAVAS OR HAWTHORN OR JABOTICABA OR "JAK FRUITS" OR "JELLY FIGS" OR KIWANO OR KIWIFRUIT OR "kiwi fruit*" OR LANGSAT OR LITCHIS OR LONGANS OR LOQUATS OR MAMEY OR MANGOES OR MANGOSTEENS OR MARULA OR MEDLARS OR medlar OR MELON OR melons OR CANTALOUPE* OR HONEYDEW OR MUSKMELONS OR MYROBALANS OR MYRTLE OR NARANJILLA OR NONI OR OLIVES OR OMIJA OR PAPAYAS OR "PASSION FRUIT*" OR passionfruit* OR PEACHES OR peach OR PEARS OR PEPINO OR PERSIMMON* OR PHALSA OR PINEAPPLE* OR PITAYOS OR PLUMS OR plum OR POMEGRANATE* OR "PRICKLY PEAR*" OR prune OR prunes OR QUINCE OR quinces OR RAMBUTAN OR rhubarb OR rockmelon* OR raspberr* OR ROSEHIP* OR SAPODILLA* OR SASKATOONS OR "saskatoon berries" OR "SEA BUCKTHORN*" OR SLOES OR SOURSOP OR SPONDIAS OR STARfruit* OR strawberr* OR tangerine* OR tangelo* OR TAMARIND* OR TOMATILLO* OR TOMATO* OR WATERMELON* OR EGUSI OR bearberr* OR soapberr* OR salmonberr* OR crowberr* OR huckleberr* OR haskap OR cranberr* OR chokecherr* OR pincherr* OR cloudberr* OR dewberr* OR elderberr* OR "partridge berries" OR artichoke* OR arugula OR Asparagus OR aubergine* OR "bamboo shoot*" OR basil OR bean OR beans OR beet OR beets OR beetroot* OR "bok choy" OR broadbean* OR broccoflower OR broccoli OR "brussel* sprout*" OR cabbage* OR caraway OR carrot* OR cauliflower* OR capsicum* OR celeriac OR celery* OR chickpea* OR chives OR cilantro OR collard* OR coriander OR corn OR courgette* OR cucumber* OR daikon OR edemame OR eggplant* OR endive OR endives OR fennel OR fiddlehead* OR garbanzos OR garlic OR ginger OR jimcama OR kale OR kohlrabi OR leek* OR legume* OR lentil OR lentils OR lettuce* OR okra OR onion* OR oregano OR peas OR parsley OR parsnip* OR pepper* OR potato* OR pumpkin* OR radish* OR radicchio OR rutabaga* OR salad OR salads OR salsify OR scallion* OR shallot* OR sorrel OR soybean* OR spinach OR squash OR sweetcorn OR "Swiss chard" OR taro OR tomatillo OR topinambur OR turnip* OR "water chestnut*" OR watercress OR yam OR yams OR zucchini* OR vegetable*)))](https://search-proquest-com.login.ezproxy.library.ualberta.ca/recentsearches.recentsearchtabview.recentsearchesgridview.scrolledrecentsearchlist.checkdbssearchlink:rerunsearch/EF475EFF1EC24C7CPQ/None?site=pqdtglobal&t:ac=RecentSearches) | 91,329 |
| S3 | [noft((animal* OR rat OR rats OR mouse OR mice OR rabbit* OR cat OR cats OR dog OR dogs OR pig OR pigs OR piglet* OR procine OR canine OR rodent* OR feline* OR sheep OR ewe OR lamb OR goat OR goats OR cow OR cows OR cattle OR bison* OR buffalo* bovine OR horse OR horses OR equine OR fish OR fishes OR "Afar Depression" OR "Great Depression" OR "inbreeding depression" OR "orange fluorescence" OR "MdPI depression" OR "fruit bat*" OR duck OR ducks OR bird OR birds OR turkey* OR chicken* OR insect* OR "fruit fly" OR "mito* depression" OR aromatherapy OR "Agent Orange" OR "Orange County" OR Cherry Valley OR "Medlars Service" OR dementia* OR alzheimer* OR "green space" or berry))](https://search-proquest-com.login.ezproxy.library.ualberta.ca/recentsearches.recentsearchtabview.recentsearchesgridview.scrolledrecentsearchlist.checkdbssearchlink:rerunsearch/F57CC2D215294A0EPQ/None?site=pqdtglobal&t:ac=RecentSearches) | [3](https://search-proquest-com.login.ezproxy.library.ualberta.ca/recentsearches.recentsearchtabview.recentsearchesgridview.scrolledrecentsearchlist.checkdbssearchlink_0:rerunsearch/F57CC2D215294A0EPQ/None?site=pqdtglobal&t:ac=RecentSearches)72,907 |
| S4 | S1 and S2 | [8](https://search-proquest-com.login.ezproxy.library.ualberta.ca/recentsearches.recentsearchtabview.recentsearchesgridview.scrolledrecentsearchlist.checkdbssearchlink_0:rerunsearch/FEA894AFE0B24F53PQ/None?site=pqdtglobal&t:ac=RecentSearches)8 |
| S5 | S4 not s3 | 38 |

**PROSPERO up to October 30, 2020**

| #1 | ((("neurotic disorder" OR hypervigilance OR agoraphobia OR "dysthymic disorder" OR anxiety OR "common mental disorders" OR "internalizing symptoms" OR "internalising symptoms" OR "mental illness" OR "clinical depression" OR "major depression" OR "mental health"))):TI | 1619 |
| --- | --- | --- |
| #2 | ("high fiber" OR "high fibre" OR "dietary fiber" OR "dietary fibre" OR FRUIT OR fruits OR ACAI OR ACKEE OR "ACTINIDIA ARGUTA" OR "AIELE FRUIT*" OR AMLA OR (APPLE* NOT (phone* OR computer* OR smartphone* OR device*)) OR APRICOTS OR apricot OR ARONIA OR avocado* OR BABACO OR BACURI OR "BAEL FRUIT" OR BANANA* OR BERRIES OR Blueberries OR barberr* OR brambleberr* OR BREADFRUIT OR BUSH BUTTER OR "CACTUS PEAR*" OR CAJA OR "CAMU CAMU" OR clementines OR GOOSEBERRIES OR CARAMBOLAS OR "CASHEW APPLE*" OR CHERIMOYA OR CHERRIES OR "CHERRY LAUREL" OR CITRUS OR ORANGES OR CITRONS OR nectarine* OR TAMURANA OR currants OR Dragonfruit* OR GALGALS OR grapes OR GRAPEFRUIT* OR "KI MIKAN" OR KUMQUAT* OR LEMONS OR lemons OR LIMES OR lingonberr* OR ORTANIQUE OR POMELOS OR TANGELO OR TANGOR OR YUZU OR COCONA OR CUPUACU OR "CURCULIGO LATIFOLIA" OR DURIAN OR FEIJOA OR FORTUNELLA OR GUAVAS OR HAWTHORN OR JABOTICABA OR "JAK FRUITS" OR "JELLY FIGS" OR KIWANO OR KIWIFRUIT OR "kiwi fruit*" OR LANGSAT OR LITCHIS OR LONGANS OR LOQUATS OR MAMEY OR MANGOES OR MANGOSTEENS OR MARULA OR MEDLARS OR medlar OR MELON OR melons OR CANTALOUPE* OR HONEYDEW OR MUSKMELONS OR MYROBALANS OR MYRTLE OR NARANJILLA OR NONI OR OLIVES OR OMIJA OR PAPAYAS OR "PASSION FRUIT*" OR passionfruit*):TI | 65 |
| #3 | (PEACHES OR peach OR PEARS OR PEPINO OR PERSIMMON* OR PHALSA OR PINEAPPLE* OR PITAYOS OR PLUMS OR plum OR POMEGRANATE* OR "PRICKLY PEAR*" OR prune OR prunes OR QUINCE OR quinces OR RAMBUTAN OR rhubarb OR rockmelon* OR raspberr* OR ROSEHIP* OR SAPODILLA* OR SASKATOONS OR "saskatoon berries" OR "SEA BUCKTHORN*" OR SLOES OR SOURSOP OR SPONDIAS OR STARfruit* OR strawberr* OR tangerine* OR tangelo* OR TAMARIND* OR TOMATILLO* OR TOMATO* OR WATERMELON* OR EGUSI OR bearberr* OR soapberr* OR salmonberr* OR crowberr* OR huckleberr* OR haskap OR cranberr* OR chokecherr* OR pincherr* OR cloudberr* OR dewberr* OR elderberr* OR "partridge berries" OR artichoke* OR arugula OR Asparagus OR aubergine* OR "bamboo shoot*" OR basil OR bean OR beans OR beet OR beets OR beetroot* OR "bok choy" OR broadbean* OR broccoflower OR broccoli OR "brussel* sprout*" OR cabbage* OR caraway OR carrot* OR cauliflower* OR capsicum* OR celeriac OR celery* OR chickpea* OR chives OR cilantro OR collard* OR coriander OR corn OR courgette* OR cucumber* OR daikon OR edemame OR eggplant* OR endive OR endives OR fennel OR fiddlehead* OR garbanzos OR garlic OR ginger OR jimcama OR kale OR kohlrabi OR leek* OR legume* OR lentil OR lentils OR lettuce* OR okra OR onion* OR oregano OR peas OR parsley OR parsnip* OR pepper* OR potato* OR pumpkin* OR radish* OR radicchio OR rutabaga* OR salad OR salads OR salsify OR scallion* OR shallot* OR sorrel OR soybean* OR spinach OR squash OR sweetcorn OR "Swiss chard" OR taro OR tomatillo OR topinambur OR turnip* OR "water chestnut*" OR watercress OR yam OR yams OR zucchini):TI | 64 |
| #4 | (vegetable*):TI | 33 |
| #5 | #2 OR #3 OR #4 | 136 |
| #6 | #1 AND #5 | 2 |

**CINAHL (EBSCO) up to October 30, 2020**

| S1 | (MH "Fruit+") OR (MH "Berries") OR (MH "Cherries") OR (MH "Citrus") or (MH "Vegetables+") | 34,631 |
| --- | --- | --- |
| S2 | TI("high fiber" OR "high fibre" OR "dietary fiber" OR "dietary fibre" OR FRUIT OR fruits OR ACAI OR ACKEE OR "ACTINIDIA ARGUTA" OR "AIELE FRUIT*" OR AMLA OR (APPLE* NOT (phone* OR computer* OR smartphone* OR device*)) OR APRICOTS OR apricot OR ARONIA OR avocado* OR BABACO OR BACURI OR "BAEL FRUIT" OR BANANA* OR BERRIES OR Blueberries OR barberr* OR brambleberr* OR BREADFRUIT OR BUSH BUTTER OR "CACTUS PEAR*" OR CAJA OR "CAMU CAMU" OR clementines OR GOOSEBERRIES OR CARAMBOLAS OR "CASHEW APPLE*" OR CHERIMOYA OR CHERRIES OR "CHERRY LAUREL" OR CITRUS OR ORANGES OR CITRONS OR nectarine* OR TAMURANA OR currants OR Dragonfruit* OR GALGALS OR grapes OR GRAPEFRUIT* OR "KI MIKAN" OR KUMQUAT* OR LEMONS OR lemons OR LIMES OR lingonberr* OR ORTANIQUE OR POMELOS OR TANGELO OR TANGOR OR YUZU OR COCONA OR CUPUACU OR "CURCULIGO LATIFOLIA" OR DURIAN OR FEIJOA OR FORTUNELLA OR GUAVAS OR HAWTHORN OR JABOTICABA OR "JAK FRUITS" OR "JELLY FIGS" OR KIWANO OR KIWIFRUIT OR "kiwi fruit*" OR LANGSAT OR LITCHIS OR LONGANS OR LOQUATS OR MAMEY OR MANGOES OR MANGOSTEENS OR MARULA OR MEDLARS OR medlar OR MELON OR melons OR CANTALOUPE* OR HONEYDEW OR MUSKMELONS OR MYROBALANS OR MYRTLE OR NARANJILLA OR NONI OR OLIVES OR OMIJA OR PAPAYAS OR "PASSION FRUIT*" OR passionfruit* OR PEACHES OR peach OR PEARS OR PEPINO OR PERSIMMON* OR PHALSA OR PINEAPPLE* OR PITAYOS OR PLUMS OR plum OR POMEGRANATE* OR "PRICKLY PEAR*" OR prune OR prunes OR QUINCE OR quinces OR RAMBUTAN OR rhubarb OR rockmelon* OR raspberr* OR ROSEHIP* OR SAPODILLA* OR SASKATOONS OR "saskatoon berries" OR "SEA BUCKTHORN*" OR SLOES OR SOURSOP OR SPONDIAS OR STARfruit* OR strawberr* OR tangerine* OR tangelo* OR TAMARIND* OR TOMATILLO* OR TOMATO* OR WATERMELON* OR EGUSI OR bearberr* OR soapberr* OR salmonberr* OR crowberr* OR huckleberr* OR haskap OR cranberr* OR chokecherr* OR pincherr* OR cloudberr* OR dewberr* OR elderberr* OR "partridge berries" OR artichoke* OR arugula OR Asparagus OR aubergine* OR "bamboo shoot*" OR basil OR bean OR beans OR beet OR beets OR beetroot* OR "bok choy" OR broadbean* OR broccoflower OR broccoli OR "brussel* sprout*" OR cabbage* OR caraway OR carrot* OR cauliflower* OR capsicum* OR celeriac OR celery* OR chickpea* OR chives OR cilantro OR collard* OR coriander OR corn OR courgette* OR cucumber* OR daikon OR edemame OR eggplant* OR endive OR endives OR fennel OR fiddlehead* OR garbanzos OR garlic OR ginger OR jimcama OR kale OR kohlrabi OR leek* OR legume* OR lentil OR lentils OR lettuce* OR okra OR onion* OR oregano OR peas OR parsley OR parsnip* OR pepper* OR potato* OR pumpkin* OR radish* OR radicchio OR rutabaga* OR salad OR salads OR salsify OR scallion* OR shallot* OR sorrel OR soybean* OR spinach OR squash OR sweetcorn OR "Swiss chard" OR taro OR tomatillo OR topinambur OR turnip* OR "water chestnut*" OR watercress OR yam OR yams OR zucchini* OR vegetable*))) | 19,574 |
| S3 | AB("high fiber" OR "high fibre" OR "dietary fiber" OR "dietary fibre" OR FRUIT OR fruits OR ACAI OR ACKEE OR "ACTINIDIA ARGUTA" OR "AIELE FRUIT*" OR AMLA OR (APPLE* NOT (phone* OR computer* OR smartphone* OR device*)) OR APRICOTS OR apricot OR ARONIA OR avocado* OR BABACO OR BACURI OR "BAEL FRUIT" OR BANANA* OR BERRIES OR Blueberries OR barberr* OR brambleberr* OR BREADFRUIT OR BUSH BUTTER OR "CACTUS PEAR*" OR CAJA OR "CAMU CAMU" OR clementines OR GOOSEBERRIES OR CARAMBOLAS OR "CASHEW APPLE*" OR CHERIMOYA OR CHERRIES OR "CHERRY LAUREL" OR CITRUS OR ORANGES OR CITRONS OR nectarine* OR TAMURANA OR currants OR Dragonfruit* OR GALGALS OR grapes OR GRAPEFRUIT* OR "KI MIKAN" OR KUMQUAT* OR LEMONS OR lemons OR LIMES OR lingonberr* OR ORTANIQUE OR POMELOS OR TANGELO OR TANGOR OR YUZU OR COCONA OR CUPUACU OR "CURCULIGO LATIFOLIA" OR DURIAN OR FEIJOA OR FORTUNELLA OR GUAVAS OR HAWTHORN OR JABOTICABA OR "JAK FRUITS" OR "JELLY FIGS" OR KIWANO OR KIWIFRUIT OR "kiwi fruit*" OR LANGSAT OR LITCHIS OR LONGANS OR LOQUATS OR MAMEY OR MANGOES OR MANGOSTEENS OR MARULA OR MEDLARS OR medlar OR MELON OR melons OR CANTALOUPE* OR HONEYDEW OR MUSKMELONS OR MYROBALANS OR MYRTLE OR NARANJILLA OR NONI OR OLIVES OR OMIJA OR PAPAYAS OR "PASSION FRUIT*" OR passionfruit* OR PEACHES OR peach OR PEARS OR PEPINO OR PERSIMMON* OR PHALSA OR PINEAPPLE* OR PITAYOS OR PLUMS OR plum OR POMEGRANATE* OR "PRICKLY PEAR*" OR prune OR prunes OR QUINCE OR quinces OR RAMBUTAN OR rhubarb OR rockmelon* OR raspberr* OR ROSEHIP* OR SAPODILLA* OR SASKATOONS OR "saskatoon berries" OR "SEA BUCKTHORN*" OR SLOES OR SOURSOP OR SPONDIAS OR STARfruit* OR strawberr* OR tangerine* OR tangelo* OR TAMARIND* OR TOMATILLO* OR TOMATO* OR WATERMELON* OR EGUSI OR bearberr* OR soapberr* OR salmonberr* OR crowberr* OR huckleberr* OR haskap OR cranberr* OR chokecherr* OR pincherr* OR cloudberr* OR dewberr* OR elderberr* OR "partrigde berries" OR artichoke* OR arugula OR Asparagus OR aubergine* OR "bamboo shoot*" OR basil OR bean OR beans OR beet OR beets OR beetroot* OR "bok choy" OR broadbean* OR broccoflower OR broccoli OR "brussel* sprout*" OR cabbage* OR caraway OR carrot* OR cauliflower* OR capsicum* OR celeriac OR celery* OR chickpea* OR chives OR cilantro OR collard* OR coriander OR corn OR courgette* OR cucumber* OR daikon OR edemame OR eggplant* OR endive OR endives OR fennel OR fiddlehead* OR garbanzos OR garlic OR ginger OR jimcama OR kale OR kohlrabi OR leek* OR legume* OR lentil OR lentils OR lettuce* OR okra OR onion* OR oregano OR peas OR parsley OR parsnip* OR pepper* OR potato* OR pumpkin* OR radish* OR radicchio OR rutabaga* OR salad OR salads OR salsify OR scallion* OR shallot* OR sorrel OR soybean* OR spinach OR squash OR sweetcorn OR "Swiss chard" OR taro OR tomatillo OR topinambur OR turnip* OR "water chestnut*" OR watercress OR yam OR yams OR zucchini* OR vegetable*))) | 35,392 |
| S4 | S1 OR S2 OR S3 | 63,270 |
| S5 | (MH "Anxiety") | 37,128 |
| S6 | (MH "Anxiety Disorders") OR (MH "Behavioral and Mental Disorders") OR (MH "Neurotic Disorders") OR (MH "Generalized Anxiety Disorder") | 10,854 |
| S7 | (MH "Depression") OR (MH "Dysthymic Disorder") OR (MH "Seasonal Affective Disorder") | 95,144 |
| S8 | TI ("mental health" or "mental illness" or anxiety or depression or depressive or "common mental disorders" or "neurotic disorder*" or "dysthymic disorder" or "seasonal affective disorder*" or "internalizing symptoms" or "internalising symptoms") OR AB ("mental health" or "mental illness" or anxiety or depression or depressive or "common mental disorders" or "neurotic disorder*" or "dysthymic disorder" or "seasonal affective disorder*" or "internalizing symptoms" or "internalising symptoms") | 231,948 |
| S9 | S5 OR S6 OR S7 OR S8 | 265,301 |
| S10 | S4 AND S9 | 1,157 |
| S11 | S4 AND S9 | 1,157 |
| S12 | S4 AND S9 | 1,157 |
| S13 | (s10 not s11) or s12 | 807 |
| S14 | (MH "Animal Diseases+") OR (MH "Animal Studies") OR (MH "Animals, Laboratory") | 110,389 |
| S15 | TI ("nursing home*" or elderly or geriatric* or woman or women or man or men or "graduate student*" or adult* or "senior citizen*" or "middle aged") | 336,132 |
| S16 | (animal* OR rat OR rats OR mouse OR mice OR rabbit* OR cat OR cats OR dog OR dogs OR pig OR pigs OR piglet* OR procine OR canine OR rodent* OR feline* OR sheep OR ewe OR lamb OR goat OR goats OR cow OR cows OR cattle OR bison* OR buffalo* bovine OR horse OR horses OR equine OR fish OR fishes OR "Afar Depression" OR "Great Depression" OR "inbreeding depression" OR "orange fluorescence" OR "MdPI depression" OR "fruit bat*" OR duck OR ducks OR bird OR birds OR turkey* OR chicken* OR insect* OR "fruit fly" OR "mito* depression" OR aromatherapy OR "Agent Orange" OR "Orange County" OR Cherry Valley OR "Medlars Service" OR dementia* OR alzheimer* OR "green space" or berry) | 361,071 |
| S17 | S14 OR S15 OR S16 | 681,904 |
| S18 | s13 not s17 | 520 |
| S19 | adult* AND (“middle school*” or pubescen* or juvenile* or teen* or youth* or “high school*” or adolesc*or prepubesc* or “pre-pubesc*” ) | 25,388 |
| S20 | adult* | 1,203,833 |
| S21 | adult* | 317,427 |
| S22 | S19 OR S21 | 323,611 |
| S23 | s20 not s22 | 880,222 |
| S24 | s18 not s23 | 495 |

**CAB Abstracts Timespan=All years (Web of Knowledge) October 30, 2020**

| #1 | TOPIC: ("neurotic disorder" or hypervigilance or agoraphobia or "dysthymic disorder" or anxiety or "common mental disorders" or cmd or "internalizing symptoms" or "internalising symptoms" or "mental illness" or "clinical depression" or "major depression" or "mental health") | [30,620](http://apps.webofknowledge.com.login.ezproxy.library.ualberta.ca/summary.do?product=CABI&doc=1&qid=47&SID=5Aziy9T7bAbLIdwzZEv&search_mode=GeneralSearch&update_back2search_link_param=yes) |
| --- | --- | --- |
| #2 | DESCRIPTORS: (depression or anxiety) | 18,200 |
| #3 | #1 or #2 | 32,671 |
| #4 | DESCRIPTORS: (vegetables OR mushrooms OR bulbous vegetables OR fruit vegetables OR leafy vegetables OR root vegetables OR stem vegetables) | 218,070 |
| #5 | DESCRIPTORS: (fruit crops OR cucurbit fruits OR melons OR watermelons OR grapes OR subtropical fruits OR subtropical small fruits OR passion fruits OR tamarillos OR subtropical tree fruits OR avocados OR carobs OR citrus fruits OR calamondins OR citrangequats OR citranges OR citremons OR citrons OR citrumelos OR clementines OR grapefruits OR kumquats OR lemons OR limes OR mandarins OR natsudaidais OR oranges OR ortaniques OR pummelos OR rough lemons OR satsumas OR sour oranges OR tangelos OR tangors OR dates OR feijoas OR longans OR loquats OR olives OR persimmons OR pomegranates OR temperate fruits OR temperate small fruits OR bilberries OR black currants OR blackberries OR blueberries OR boysenberries OR cranberries OR gooseberries OR kiwifruits OR loganberries OR raspberries OR red currants OR strawberries OR tayberries OR white currants OR temperate tree fruits OR tropical fruits OR tropical small fruits OR bananas OR pineapples OR tropical tree fruits OR small fruits OR subtropical small fruits OR temperate small fruits OR tropical small fruits OR tree fruits OR subtropical tree fruits OR temperate tree fruits OR tropical tree fruits) | 406,040 |
| #6 | 567,984  TITLE: ((("high fiber" or "high fibre" or "dietary fiber" or "dietary fibre" or FRUIT or fruits or ACAI or ACKEE or "ACTINIDIA ARGUTA" or "AIELE FRUIT*" or AMLA or (APPLE* not (phone* or computer* or smartphone* or device*)) or APRICOTS or apricot or ARONIA or avocado* or BABACO or BACURI or "BAEL FRUIT" or BANANA* or BERRIES or Blueberries or barberr* or brambleberr* or BREADFRUIT or BUSH BUTTER or "CACTUS PEAR*" or CAJA or "CAMU CAMU" or clementines or GOOSEBERRIES or CARAMBOLAS or "CASHEW APPLE*" or CHERIMOYA or CHERRIES or "CHERRY LAUREL" or CITRUS or ORANGES or CITRONS or nectarine* or TAMURANA or currants or Dragonfruit* or GALGALS or grapes or GRAPEFRUIT* or "KI MIKAN" or KUMQUAT* or LEMONS or lemons or LIMES or lingonberr* or ORTANIQUE or POMELOS or TANGELO or TANGOR or YUZU or COCONA or CUPUACU or "CURCULIGO LATIFOLIA" or DURIAN or FEIJOA or FORTUNELLA or GUAVAS or HAWTHORN or JABOTICABA or "JAK FRUITS" or "JELLY FIGS" or KIWANO or KIWIFRUIT or "kiwi fruit*" or LANGSAT or LITCHIS or LONGANS or LOQUATS or MAMEY or MANGOES or MANGOSTEENS or MARULA or MEDLARS or medlar or MELON or melons or CANTALOUPE* or HONEYDEW or MUSKMELONS or MYROBALANS or MYRTLE or NARANJILLA or NONI or OLIVES or OMIJA or PAPAYAS or "PASSION FRUIT*" or passionfruit* or PEACHES or peach or PEARS or PEPINO or PERSIMMON* or PHALSA or PINEAPPLE* or PITAYOS or PLUMS or plum or POMEGRANATE* or "PRICKLY PEAR*" or prune or prunes or QUINCE or quinces or RAMBUTAN or rhubarb or rockmelon* or raspberr* or ROSEHIP* or SAPODILLA* or SASKATOONS or "saskatoon berr*" or "SEA BUCKTHORN*" or SLOES or SOURSOP or SPONDIAS or STARfruit* or strawberr* or tangerine* or tangelo* or TAMARIND* or TOMATILLO* or TOMATO* or WATERMELON* or EGUSI or bearberr* or soapberr* or salmonberr* or crowberr* or huckleberr* or haskap or cranberr* or chokecherr* or pincherr* or cloudberr* or dewberr* or elderberr* or "partridge berr*")) OR TOPIC: ((Artichoke* or arugula or Asparagus or aubergine* or "bamboo shoot*" or basil or bean or beans or beet or beets or beetroot* or "bok choy" or broadbean* or broccoflower or broccoli or "brussel* sprout*" or cabbage* or caraway or carrot* or cauliflower* or capsicum* or celeriac or celery* or chickpea* or chives or cilantro or collard* or coriander or corn or courgette* or cucumber* or daikon or edemame or eggplant* or endive or endives or fennel or fiddlehead* or garbanzos or garlic or ginger or greens or jimcama or kale or kohlrabi or leek* or legume* or lentil or lentils or lettuce* or okra or onion* or oregano or peas or parsley or parsnip* or pepper* or potato* or pumpkin* or radish* or radicchio or rutabaga* or salad or salads or salsify or scallion* or shallot* or sorrel or soybean* or spinach or squash or sweetcorn or "Swiss chard" or taro or tomatillo or topinambur or turnip* or "water chestnut*" or watercress or yam or yams or zucchini* or vegetable*)) OR DESCRIPTORS: (VEGETABLES OR DRIED PEAS OR DRIED VEGETABLES OR VEGETABLES SPECIFIC OR ALLIUM OR AMARANTH LEAVES OR ANU OR ARRACACHA OR ARROWHEAD OR ARTICHOKES OR ASPARAGUS OR AUBERGINES OR AVOCADOS OR BAMBOO OR BAOBAB OR BEETS OR BITTER LEAF OR BLACK SALSIFY OR BORAGE OR BOXTHORN OR BRASSICA OR BROCCOLI OR BRUSSELS SPROUTS OR BURDOCK OR BUTTERBUR OR CABBAGES OR CANNA OR CAPSICUMS OR CARDOONS OR CARROTS OR CASSAVA OR CAULIFLOWERS OR CELERIAC OR CELERY OR CEYLON SPINACH OR CHAYOTE OR CHICORY (VEGETABLES) OR CHINESE CABBAGES OR CHINESE CHIVES OR CHINESE WATER CHESTNUTS OR CHUFA NUTS OR COCOYAMS OR CORCHORUS OLITORIUS OR COURGETTES OR CRESS OR CRUCIFERAE OR CUCUMBERS OR CUCURBITACEAE OR CURCUMA OR DANDELIONS OR EDIBLE FLOWERS OR ELEPHANT YAMS OR ENDIVES OR ERUCA SATIVA OR EWEDU OR EXOTIC VEGETABLES OR FENNEL OR FENUGREEK OR FUNGI EDIBLE OR GHERKINS OR GLOBE ARTICHOKES OR GODULBAEGI OR GOURDS OR GREEN VEGETABLES OR HOUTTUYNIA CORDATA OR INDIAN SPINACH OR JERUSALEM ARTICHOKES OR KALE OR KOHLRABI OR KOMATSUNA OR KUDZU OR LEAFY VEGETABLES OR LEEKS OR LEGUMES OR LETTUCES OR LILY BULBS OR LOTUS ROOTS OR MACA OR MARROWS OR MUSTARD GREENS OR NETTLES OR OCA OR OKRA OR ONIONS OR PALM HEARTS OR PARSNIPS OR PERILLA OR POTATOES OR PUMPKINS OR PURSLANE OR RADISHES OR RAKKYO OR RHUBARB OR ROCKET OR ROOT CROPS OR ROOT VEGETABLES OR SALAD VEGETABLES OR SALSIFY OR SCALLION OR SEA KALE OR SESBANIA OR SHALLOTS OR SOLANUM OR SPINACH OR SPROUTS OR SQUASHES OR SWEDES OR SWEET POTATOES OR SWEETCORN OR SWISS CHARD OR TUBERS OR TURNIPS OR ULLUCO OR VINE LEAVES OR WATER CHESTNUTS OR WATER DROPWORT OR WATER SPINACH OR WATERCRESS OR WELSH ONIONS OR WILD CABBAGE OR WILD VEGETABLES OR YACON OR YAMS OR ZEDOARY))  Indexes=CAB Abstracts Timespan=All years | 582,824 |
| #7 | #6 OR #5 OR #4 | 894,183 |
| #8 | #7 AND #3 | 978 |
| #9 | DESCRIPTORS: (laboratory animals) | 132,386 |
| #10 | DESCRIPTORS: (animal models) | 246,288 |
| #11 | TOPIC: (rat OR rats OR mouse OR mice OR rabbit* OR cat OR cats OR dog OR dogs OR pig OR pigs OR piglet* OR procine OR canine OR rodent* OR feline* OR sheep OR ewe OR lamb OR goat OR goats OR cow OR cows OR cattle OR bison* OR buffalo* bovine OR horse OR horses OR equine OR fish OR fishes OR "Afar Depression" OR "Great Depression" OR "inbreeding depression" OR "orange fluorescence" OR "MdPI depression" OR "fruit bat*" OR duck OR ducks OR bird OR birds OR turkey* OR chicken* OR insect* OR "fruit fly" OR "mito* depression" OR aromatherapy OR "Agent Orange" OR "Orange County" OR Cherry Valley OR "Medlars Service" OR dementia* OR alzheimer* OR "green space") | 3,903,342 |
| #12 | #11 OR #10 OR #9 | 3,922,324 |
| #13 | #8 not #12 | 393 |

**Cochrane Library (Wiley) April 1996 - October 30 2020**

| **#1** | MeSH descriptor: [Fruit] explode all trees | **2535** |
| --- | --- | --- |
| **#2** | MeSH descriptor: [Vegetables] explode all trees | **1815** |
| **#3** | ("high fiber" or "high fibre" or "dietary fiber" or "dietary fibre" or FRUIT or fruits or ACAI or ACKEE or "ACTINIDIA ARGUTA" or "AIELE FRUIT*" or AMLA or (APPLE* not (phone* or computer* or smartphone* or device*)) or APRICOTS or apricot or ARONIA or avocado* or BABACO or BACURI or "BAEL FRUIT" or BANANA* or BERRIES or Blueberries or barberr* or brambleberr* or BREADFRUIT or BUSH BUTTER or "CACTUS PEAR*" or CAJA or "CAMU CAMU" or clementines or GOOSEBERRIES or CARAMBOLAS or "CASHEW APPLE*" or CHERIMOYA or CHERRIES or "CHERRY LAUREL" or CITRUS or ORANGES or CITRONS or nectarine* or TAMURANA or currants or Dragonfruit* or GALGALS or grapes or GRAPEFRUIT* or "KI MIKAN" or KUMQUAT* or LEMONS or lemons or LIMES or lingonberr* or ORTANIQUE or POMELOS or TANGELO or TANGOR or YUZU or COCONA or CUPUACU or "CURCULIGO LATIFOLIA" or DURIAN or FEIJOA or FORTUNELLA or GUAVAS or HAWTHORN or JABOTICABA or "JAK FRUITS" or "JELLY FIGS" or KIWANO or KIWIFRUIT or "kiwi fruit*" or LANGSAT or LITCHIS or LONGANS or LOQUATS or MAMEY or MANGOES or MANGOSTEENS or MARULA or MEDLARS or medlar or MELON or melons or CANTALOUPE* or HONEYDEW or MUSKMELONS or MYROBALANS or MYRTLE or NARANJILLA or NONI or OLIVES or OMIJA or PAPAYAS or "PASSION FRUIT*" or passionfruit* or PEACHES or peach or PEARS or PEPINO or PERSIMMON* or PHALSA or PINEAPPLE* or PITAYOS or PLUMS or plum or POMEGRANATE* or "PRICKLY PEAR*" or prune or prunes or QUINCE or quinces or RAMBUTAN or rhubarb or rockmelon* or raspberr* or ROSEHIP* or SAPODILLA* or SASKATOONS or "saskatoon berr*" or "SEA BUCKTHORN*" or SLOES or SOURSOP or SPONDIAS or STARfruit* or strawberr* or tangerine* or tangelo* or TAMARIND* or TOMATILLO* or TOMATO* or WATERMELON* or EGUSI or bearberr* or soapberr* or salmonberr* or crowberr* or huckleberr* or haskap or cranberr* or chokecherr* or pincherr* or cloudberr* or dewberr* or elderberr* or "partridge berr*" OR Artichoke* or arugula or Asparagus or aubergine* or "bamboo shoot*" or basil or bean or beans or beet or beets or beetroot* or "bok choy" or broadbean* or broccoflower or broccoli or "brussel* sprout*" or cabbage* or caraway or carrot* or cauliflower* or capsicum* or celeriac or celery* or chickpea* or chives or cilantro or collard* or coriander or corn or courgette* or cucumber* or daikon or edemame or eggplant* or endive or endives or fennel or fiddlehead* or garbanzos or garlic or ginger or greens or jimcama or kale or kohlrabi or leek* or legume* or lentil or lentils or lettuce* or okra or onion* or oregano or peas or parsley or parsnip* or pepper* or potato* or pumpkin* or radish* or radicchio or rutabaga* or salad or salads or salsify or scallion* or shallot* or sorrel or soybean* or spinach or squash or sweetcorn or "Swiss chard" or taro or tomatillo or topinambur or turnip* or "water chestnut*" or watercress or yam or yams or zucchini* or vegetable*):ti,kw | **15146** |
| **#4** | #1 or #2 or #3 | **15923** |
| **#5** | MeSH descriptor: [Anxiety] explode all trees | **7510** |
| **#6** | MeSH descriptor: [Anxiety Disorders] this term only | **3334** |
| **#7** | MeSH descriptor: [Depression] this term only | **10762** |
| **#8** | MeSH descriptor: [Depressive Disorder, Major] explode all trees | **4393** |
| **#9** | hypervigilance or agoraphobia or "dysthymic disorder" or anxiety or "common mental disorders" o "internalizing symptoms" or "internalising symptoms" or "mental illness" or "clinical depression" or "major depression" or "mental health":ti,ab,kw | **71098** |
| **#10** | #5 or #6 or #7 or #8 or #9 | **78087** |
| **#11** | #4 and #10 | **296** |
| **#12** | (rat OR rats OR mouse OR mice OR rabbit* OR cat OR cats OR dog OR dogs OR pig OR pigs OR piglet* OR procine OR canine OR rodent* OR feline* OR sheep OR ewe OR lamb OR goat OR goats OR cow OR cows OR cattle OR bison* OR buffalo* bovine OR horse OR horses OR equine OR fish OR fishes OR "Afar Depression" OR "Great Depression" OR "inbreeding depression" OR "orange fluorescence" OR "MdPI depression" OR "fruit bat*" OR duck OR ducks OR bird OR birds OR turkey* OR chicken* OR insect* OR "fruit fly" OR "mito* depression" OR aromatherapy OR "Agent Orange" OR "Orange County" OR Cherry Valley OR "Medlars Service" OR dementia* OR alzheimer* OR "green space" or animal*) | **98475** |
| **#13** | #11 not #12 | **202** |
| **#14** | adult* or woman or man or elderly or "senior citizen*" or "nursing home*" or women or men or toddler* or preschooler* | **747160** |
| **#15** | #13 not #14 | **65** |

**Supplementary File 2**

**PRISMA chart**

3944 studies imported for screening

903 duplicates removed

3041 studies screened

2924 studies irrelevant

117 Full-text studies assessed for eligibility

105 studies excluded

67 Wrong population

20 Wrong exposure

9 Wrong outcomes

3 Duplicate

3 Review/letter to the editor

3 Unable to locate full-text paper

12 studies included in the analysis

**Supplementary File 3**

Table S1. Results of the studies (n=12) included in the systematic review (systematic review, all countries, up to 2020).

|  | Statistical method | Subgroup analysis | Comparator | Effect measure | Effect size (95% CI) | Covariates adjusted for |
| --- | --- | --- | --- | --- | --- | --- |
| Depression | | | | | | |
| *Fruit* | | | | | | |
| Arat 2017[23] | binomial logistic regression | Botswana | high (≥1 time/ day) vs. low (6 times or less during the past 30 days) | OR | 1.01 (0.98; 1.04) | age, gender, bullying, close friends, parental control, hunger (proxy for social status) |
|  |  | Kenya |  |  | 1.08 (1.07; 1.09) |  |
|  |  | Seychelles |  |  | 1.47 (1.27; 1.71) |  |
|  |  | Uganda |  |  | 1.03 (1.01; 1.04) |  |
|  |  | Tanzania |  |  | 1.33 (1.29; 1.38) |  |
|  |  | Zambia |  |  | 1.17 (1.15; 1.2) |  |
| Arat 2015[24] | simple logistic regression | Asian American | high (≥1 time/ day) vs. low (6 times or less during the past 7 days) | OR | 1.29 (0.75; 2.21) | stratified by ethnicity; no other covariates controlled for in data analysis |
|  |  | African American |  |  | 1.14 (0.96; 1.34) |  |
|  |  | Caucasian |  |  | 1.1 (0.9; 1.33) |  |
| Hoare et al. 2019[25] | multiple logistic regression | N/A | consumers (i.e., consumed F/V according to 24hr recall) vs. non-consumers | OR | 1.10 (0.9; 1.33) | unadjusted |
|  |  |  |  |  | 1.08 (0.9; 1.30) | age, sex |
|  |  |  |  |  | 1.07 (0.88; 1.30) | age, sex, school socioeconomic level |
|  |  |  |  |  | 1.07 (0.89; 1.28) | age, sex, school socioeconomic level, weight status |
| Hoare et al. 2018[27] | multiple logistic regression | females | 1 time/week vs. none | OR | 0.59 (0.43; 0.81) | unadjusted |
|  |  |  | ≥2 times/week vs. none |  | 0.54 (0.40; 0.73) |  |
|  |  | males | 1 time/week vs. none |  | 0.48 (0.33; 0.70) |  |
|  |  |  | ≥2 times/week vs. none |  | 0.50 (0.35; 0.71) |  |
|  |  | females | 1 time/week vs. none |  | 0.68 (0.49; 0.95) | stratified by gender; adjusted for age, household income, ethnicity, physical activity, BMI in data analysis |
|  |  |  | ≥2 times/week vs. none |  | 0.62 (0.45; 0.85) |  |
|  |  | males | 1 time/week vs. none |  | 0.53 (0.36; 0.77) |  |
|  |  |  | ≥2 times/week vs. none |  | 0.55 (0.38; 0.8) |  |
| Hong and Peltzer 2018[28] | multivariate logistic regression | N/A | “1-2 times/week” vs. “I did not eat” | OR | 0.88 (0.83; 0.94) | age, sex, socioeconomic status, school level, school types, BMI, physical activity, and substance use |
|  |  |  | “3-4 times/week” vs. “I did not eat” |  | 0.83 (0.77; 0.88) |  |
|  |  |  | “5-6 times/week” vs. “I did not eat” |  | 0.83 (0.77; 0.9) |  |
|  |  |  | “once/day” vs. “I did not eat” |  | 0.86 (0.79; 0.92) |  |
|  |  |  | “twice/day” vs. “I did not eat” |  | 0.86 (0.78; 0.94) |  |
|  |  |  | “≥3 times/day” vs. “I did not eat” |  | 1.05 (0.95; 1.17) |  |
| Kim et al. 2015[34] | multivariable logistic regression | N/A | 7.2-22.9 servings/week vs. 7.2 or lower | OR | 0.56 (0.34; 0.94) | energy intake, menstrual regularity |
|  |  |  | >22.9 servings/week vs. 7.2 or lower |  | 0.63 (0.37; 1.06) |  |
| Ming-wei Liu et al.[29] | multivariable logistic regression | Saint Lucia | <2 times/day vs. none | OR | 0.92 (0.49; 1.75) | Age, sex, physical activity, sedentary behaviour, being bullied, smoking status, alcohol intake |
|  |  |  | ≥2 times/day vs. none |  | 0.91 (0.43; 1.91) |  |
|  |  | Egypt | <2 times/day vs. none |  | 0.57 (0.31; 1.02) | Age, sex, physical activity, sedentary behaviour, being bullied |
|  |  |  | ≥2 times/day vs. none |  | 0.57 (0.31; 1.03) |  |
|  |  | Saint Vincent and Grenadines | <2 times/day vs. none |  | 1.28 (0.65; 2.52) | Age, sex, physical activity, sedentary behaviour, being bullied, smoking status, alcohol intake |
|  |  |  | ≥2 times/day vs. none |  | 1.29 (0.67; 2.50) |  |
|  |  | Djibouti | <2 times/day vs. none |  | 0.98 (0.64; 1.55) | Age, sex, physical activity, sedentary behaviour, being bullied, smoking status |
|  |  |  | ≥2 times/day vs. none |  | 0.87 (0.53; 1.45) |  |
|  |  | Morocco | <2 times/day vs. none |  | 1.01 (0.46; 2.20) | Age, sex, physical activity, sedentary behaviour, being bullied, smoking status, alcohol intake |
|  |  |  | ≥2 times/day vs. none |  | 0.75 (0.36; 1.58) |  |
|  |  | Myanmar | <2 times/day vs. none |  | 1.08 (0.66; 1.77) | Age, sex, physical activity, sedentary behaviour, being bullied, smoking status, alcohol intake |
|  |  |  | ≥2 times/day vs. none |  | 1.12 (0.62; 2.04) |  |
|  |  | Zambia | <2 times/day vs. none |  | 1.52 (0.92; 2.52) | Age, sex, physical activity, sedentary behaviour, being bullied, alcohol intake |
|  |  |  | ≥2 times/day vs. none |  | 1.26 (0.79; 2.00) |  |
|  |  | United Republic of Tanzania | <2 times/day vs. none |  | 0.74 (0.51; 1.06) | Age, sex, physical activity, sedentary behaviour, being bullied, smoking status, alcohol intake |
|  |  |  | ≥2 times/day vs. none |  | 0.67 (0.51; 0.87) |  |
|  |  | Venezuela | <2 times/day vs. none |  | 0.81 (0.58; 1.12) | Age, sex, physical activity, sedentary behaviour, being bullied, alcohol intake |
|  |  |  | ≥2 times/day vs. none |  | 0.89 (0.65; 1.21) |  |
|  |  | Grenada | <2 times/day vs. none |  | 0.82 (0.42; 1.60) | Age, sex, physical activity, sedentary behaviour, being bullied, smoking status, alcohol intake |
|  |  |  | ≥2 times/day vs. none |  | 0.59 (0.33; 1.07) |  |
|  |  | Lebanon | <2 times/day vs. none |  | 0.81 (0.51; 1.27) | Age, sex, being bullied, alcohol intake |
|  |  |  | ≥2 times/day vs. none |  | 0.82 (0.52; 1.30) |  |
|  |  | China | <2 times/day vs. none |  | 0.63 (0.46; 0.86) | Age, sex, physical activity, sedentary behaviour, being bullied, smoking status, alcohol intake |
|  |  |  | ≥2 times/day vs. none |  | 0.62 (0.46; 0.83) |  |
|  |  | Indonesia | <2 times/day vs. none |  | 0.61 (0.36; 1.005) | Age, sex, physical activity, sedentary behaviour, being bullied, smoking status, alcohol intake |
|  |  |  | ≥2 times/day vs. none |  | 0.57 (0.35; 0.92) |  |
|  |  | Thailand | <2 times/day vs. none |  | 0.54 (0.31; 0.97) | Age, sex, physical activity, sedentary behaviour, being bullied, smoking status, alcohol intake |
|  |  |  | ≥2 times/day vs. none |  | 0.56 (0.37; 0.83) |  |
|  |  | Uganda | <2 times/day vs. none |  | 0.98 (0.80; 1.20) | Age, sex, physical activity, sedentary behaviour, being bullied, smoking status, alcohol intake |
|  |  |  | ≥2 times/day vs. none |  | 0.93 (0.72; 1.21) |  |
|  |  | Tunisia | <2 times/day vs. none |  | 0.67 (0.41; 1.07) | Age, sex, physical activity, sedentary behaviour, being bullied, smoking status |
|  |  |  | ≥2 times/day vs. none |  | 0.71 (0.43; 1.20) |  |
|  |  | Botswana | <2 times/day vs. none |  | 0.87 (0.57; 1.32) | Age, sex, physical activity, sedentary behaviour, being bullied, smoking status, alcohol intake |
|  |  |  | ≥2 times/day vs. none |  | 0.70 (0.47; 1.04) |  |
|  |  | Sri Lanka | <2 times/day vs. none |  | 0.77 (0.55; 1.07) | Age, sex, physical activity, sedentary behaviour, being bullied |
|  |  |  | ≥2 times/day vs. none |  | 0.69 (0.46; 1.02) |  |
|  |  | India | <2 times/day vs. none |  | 0.69 (0.53; 0.90) | Age, sex, physical activity, sedentary behaviour, smoking status |
|  |  |  | ≥2 times/day vs. none |  | 0.67 (0.51; 0.89) |  |
|  |  | Seychelles | <2 times/day vs. none |  | 0.74 (0.62; 0.87) | Age, sex, physical activity, sedentary behaviour, being bullied, smoking status, alcohol intake |
|  |  |  | ≥2 times/day vs. none |  | 0.61 (0.52; 0.71) |  |
|  |  | Guyana | <2 times/day vs. none |  | 0.81 (0.46; 1.42) | Age, sex, physical activity, sedentary behaviour, being bullied, smoking status, alcohol intake |
|  |  |  | ≥2 times/day vs. none |  | 0.64 (0.31; 1.33) |  |
|  |  | Ecuador | <2 times/day vs. none |  | 0.76 (0.56; 1.03) | Age, sex, physical activity, sedentary behaviour, being bullied, smoking status, alcohol intake |
|  |  |  | ≥2 times/day vs. none |  | 0.71 (0.53; 0.94) |  |
|  |  | Jordan | <2 times/day vs. none |  | 0.54 (0.33; 0.88) | Age, sex, physical activity, sedentary behaviour, being bullied, smoking status |
|  |  |  | ≥2 times/day vs. none |  | 0.51 (0.29; 0.91) |  |
|  |  | Argentina | <2 times/day vs. none |  | 0.83 (0.51; 1.34) | Age, sex, physical activity, sedentary behaviour, being bullied, smoking status, alcohol intake |
|  |  |  | ≥2 times/day vs. none |  | 1.12 (0.75; 1.69) |  |
|  |  | Kenya | <2 times/day vs. none |  | 0.79 (0.52; 1.22) | Age, sex, physical activity, sedentary behaviour, being bullied, smoking status, alcohol intake |
|  |  |  | ≥2 times/day vs. none |  | 0.90 (0.61; 1.35) |  |
| Park et al. 2018[30] | multiple logistic regression | N/A | high (once or more every day) vs. low-frequency consumption group (less than once per day) | OR | 1.03 (0.99; 1.08) | sex, school grade, residential area, socioeconomic status, and other dietary behaviours (skipping breakfast, milk 2 or more times/day, fast food 3 times or more per week, soft drinks 3 times or more per week, vegetables (3 times or more per day)) |
| *Vegetables* |  |  |  |  |  |  |
| Arat 2017[23] | binomial logistic regression | Botswana | high (≥1 time/ day) vs. low (6 times or less during the past 7 days) | OR | 1.06 (1.02; 1.09) | age, gender, bullying, close friends, parental control, hunger (proxy for social status) |
|  |  | Kenya |  |  | 1.11 (1.1; 1.12) |  |
|  |  | Seychelles |  |  | 1.14 (0.97; 1.33) |  |
|  |  | Uganda |  |  | 1.04 (1.03; 1.06) |  |
|  |  | Tanzania |  |  | 1.14 (1.1; 1.19) |  |
|  |  | Zambia |  |  | 1.17 (1.14; 1.19) |  |
| Arat 2015[24] | simple logistic regression | Asian American | high (≥1 time/ day) vs. low (6 times or less during the past 7 days) | OR | 1.09 (0.68; 1.74) | stratified by ethnicity; no other covariates controlled for in data analysis |
|  |  | African American |  |  | 0.9 (0.78; 1.04) |  |
|  |  | Caucasian |  |  | 0.98 (0.84; 1.14) |  |
| Hoare et al. 2019[25] | multiple logistic regression | N/A | consumers (i.e., consumed F/V according to 24hr recall) vs. non-consumers | OR | 1.04 (0.87; 1.24) | unadjusted |
|  |  |  |  |  | 1.00 (0.83; 1.21) | age, sex |
|  |  |  |  |  | 0.99 (0.79; 1.25) | age, sex, school socioeconomic level |
|  |  |  |  |  | 1 (0.84; 1.2) | age, sex, school socioeconomic level, weight status |
| Hoare et al. 2018[27] | multiple logistic regression | females | 1 time/week vs. none | OR | 0.65 (0.49; 0.85) | unadjusted |
|  |  |  | ≥2 times/week vs. none |  | 0.54 (0.39; 0.73) |  |
|  |  | males | 1 time/week vs. none |  | 0.79 (0.56; 1.11) |  |
|  |  |  | ≥2 times/week vs. none |  | 0.79 (0.54; 1.16) |  |
|  |  | females | 1 time/week vs. none |  | 0.69 (0.52; 0.93) | stratified by gender; adjusted for age, household income, ethnicity, physical activity, BMI in data analysis |
|  |  |  | ≥2 times/week vs. none |  | 0.64 (0.46; 0.88) |  |
|  |  | males | 1 time/week vs. none |  | 0.8 (0.56; 1.13) |  |
|  |  |  | ≥2 times/week vs. none |  | 0.87 (0.59; 1.28) |  |
| Hong and Peltzer 2018[28] | multivariate logistic regression | N/A | “1-2 times/week” vs. “I did not eat” | OR | 0.9 (0.82; 1) | age, sex, socioeconomic status, school level, school types, Body Mass Index, physical activity, and substance use |
|  |  |  | “3-4 times/week” vs. “I did not eat” |  | 0.79 (0.72; 0.87) |  |
|  |  |  | “5-6 times/week” vs. “I did not eat” |  | 0.8 (0.72; 0.88) |  |
|  |  |  | “once/day” vs. “I did not eat” |  | 0.84 (0.76; 0.93) |  |
|  |  |  | “twice/day” vs. “I did not eat” |  | 0.78 (0.7; 0.86) |  |
|  |  |  | “≥3 times/day” vs. “I did not eat” |  | 0.83 (0.75; 0.92) |  |
| Kim et al. 2015[34] | multivariable logistic regression | N/A | 7.2-22.9 servings/week compared to 7.2 or lower | OR | 1 (0.62; 1.61) | energy intake, menstrual regularity |
|  |  |  | >22.9 servings/week compared to 7.2 or lower |  | 0.61 (0.35; 1.04) |  |
| Ming-wei Liu et al.[29] | multivariable logistic regression | Saint Lucia | <3 times/day vs none | OR | 0.83 (0.40; 1.74) | Age, sex, physical activity, sedentary behaviour, being bullied, smoking status, alcohol intake |
|  |  |  | ≥3 times/day vs none |  | 1.27 (0.50; 3.21) |  |
|  |  | Egypt | <3 times/day vs none |  | 0.59 (0.47; 0.76) | Age, sex, physical activity, sedentary behaviour, being bullied |
|  |  |  | ≥3 times/day vs none |  | 0.82 (0.49; 1.35) |  |
|  |  | Saint Vincent and Grenadines | <3 times/day vs none |  | 0.71 (0.42; 1.19) | Age, sex, physical activity, sedentary behaviour, being bullied, smoking status, alcohol intake |
|  |  |  | ≥3 times/day vs none |  | 0.76 (0.43; 1.35) |  |
|  |  | Djibouti | <3 times/day vs none |  | 0.99 (0.57; 1.72) | Age, sex, physical activity, sedentary behaviour, being bullied, smoking status |
|  |  |  | ≥3 times/day vs none |  | 1.17 (0.73; 1.89) |  |
|  |  | Morocco | <3 times/day vs none |  | 0.43 (0.12; 1.56) | Age, sex, physical activity, sedentary behaviour, being bullied, smoking status, alcohol intake |
|  |  |  | ≥3 times/day vs none |  | 0.60 (0.16; 2.25) |  |
|  |  | Myanmar | <3 times/day vs none |  | 0.53 (0.17; 1.63) | Age, sex, physical activity, sedentary behaviour, being bullied, smoking status, alcohol intake |
|  |  |  | ≥3 times/day vs none |  | 0.69 (0.21; 2.24) |  |
|  |  | Zambia | <3 times/day vs none |  | 0.86 (0.47; 1.55) | Age, sex, physical activity, sedentary behaviour, being bullied, alcohol intake |
|  |  |  | ≥3 times/day vs none |  | 1.10 (0.60; 2.01) |  |
|  |  | United Republic of Tanzania | <3 times/day vs none |  | 0.43 (0.26; 0.69) | Age, sex, physical activity, sedentary behaviour, being bullied, smoking status, alcohol intake |
|  |  |  | ≥3 times/day vs none |  | 0.56 (0.31; 0.99) |  |
|  |  | Venezuela | <3 times/day vs none |  | 0.79 (0.59; 1.06) | Age, sex, physical activity, sedentary behaviour, being bullied, alcohol intake |
|  |  |  | ≥3 times/day vs none |  | 0.73 (0.55; 0.97) |  |
|  |  | Grenada | <3 times/day vs none |  | 0.68 (0.45; 1.03) | Age, sex, physical activity, sedentary behaviour, being bullied, smoking status, alcohol intake |
|  |  |  | ≥3 times/day vs none |  | 0.85 (0.54; 1.35) |  |
|  |  | Lebanon | <3 times/day vs none |  | 0.55 (0.42; 0.73) | Age, sex, being bullied, alcohol intake |
|  |  |  | ≥3 times/day vs none |  | 0.57 (0.41; 0.78) |  |
|  |  | China | <3 times/day vs none |  | 0.42 (0.21; 0.87) | Age, sex, physical activity, sedentary behaviour, being bullied, smoking status, alcohol intake |
|  |  |  | ≥3 times/day vs none |  | 0.44 (0.22; 0.88) |  |
|  |  | Indonesia | <3 times/day vs none |  | 0.56 (0.30; 1.04) | Age, sex, physical activity, sedentary behaviour, being bullied, smoking status, alcohol intake |
|  |  |  | ≥3 times/day vs none |  | 0.58 (0.30; 1.15) |  |
|  |  | Thailand | <3 times/day vs none |  | 1.19 (0.53; 2.67) | Age, sex, physical activity, sedentary behaviour, being bullied, smoking status, alcohol intake |
|  |  |  | ≥3 times/day vs none |  | 1.13 (0.51; 2.51) |  |
|  |  | Uganda | <3 times/day vs none |  | 0.85 (0.66; 1.10) | Age, sex, physical activity, sedentary behaviour, being bullied, smoking status, alcohol intake |
|  |  |  | ≥3 times/day vs none |  | 0.85 (0.57; 1.28) |  |
|  |  | Tunisia | <3 times/day vs none |  | 0.86 (0.64; 1.16) | Age, sex, physical activity, sedentary behaviour, being bullied, smoking status |
|  |  |  | ≥3 times/day vs none |  | 0.82 (0.60; 1.14) |  |
|  |  | Botswana | <3 times/day vs none |  | 0.80 (0.57; 1.13) | Age, sex, physical activity, sedentary behaviour, being bullied, smoking status, alcohol intake |
|  |  |  | ≥3 times/day vs none |  | 0.90 (0.60; 1.35) |  |
|  |  | Sri Lanka | <3 times/day vs none |  | 0.53 (0.34; 0.82) | Age, sex, physical activity, sedentary behaviour, being bullied |
|  |  |  | ≥3 times/day vs none |  | 0.55 (0.31; 0.99) |  |
|  |  | India | <3 times/day vs none |  | 1.14 (0.65; 1.99) | Age, sex, physical activity, sedentary behaviour, smoking status |
|  |  |  | ≥3 times/day vs none |  | 1.03 (0.62; 1.71) |  |
|  |  | Seychelles | <3 times/day vs none |  | 1.00 (0.86; 1.16) | Age, sex, physical activity, sedentary behaviour, being bullied, smoking status, alcohol intake |
|  |  |  | ≥3 times/day vs none |  | 0.88 (0.76; 1.03) |  |
|  |  | Guyana | <3 times/day vs none |  | 0.63 (0.26; 1.52) | Age, sex, physical activity, sedentary behaviour, being bullied, smoking status, alcohol intake |
|  |  |  | ≥3 times/day vs none |  | 0.71 (0.28; 1.78) |  |
|  |  | Ecuador | <3 times/day vs none |  | 0.69 (0.58; 0.84) | Age, sex, physical activity, sedentary behaviour, being bullied, smoking status, alcohol intake |
|  |  |  | ≥3 times/day vs none |  | 0.59 (0.48; 0.74) |  |
|  |  | Jordan | <3 times/day vs none |  | 1.01 (0.57; 0.81) | Age, sex, physical activity, sedentary behaviour, being bullied, smoking status |
|  |  |  | ≥3 times/day vs none |  | 0.75 (0.38; 1.48) |  |
|  |  | Argentina | <3 times/day vs none |  | 0.78 (0.57; 1.08) | Age, sex, physical activity, sedentary behaviour, being bullied, smoking status, alcohol intake |
|  |  |  | ≥3 times/day vs none |  | 0.50 (0.32; 0.77) |  |
|  |  | Kenya | <3 times/day vs none |  | 0.82 (0.47; 1.44) | Age, sex, physical activity, sedentary behaviour, being bullied, smoking status, alcohol intake |
|  |  |  | ≥3 times/day vs none |  | 0.76 (0.43; 1.34) |  |
| Park et al. 2018[30] | multiple logistic regression | N/A | high (once or more every day) vs. low-frequency consumption group (less than once per day) | OR | 1.01 (0.97; 1.06) | sex, school grade, residential area, socioeconomic status, and other dietary behaviours (skipping breakfast, milk 2 or more times/day, fast food 3 times or more per week, soft drinks 3 times or more per week, vegetables (3 times or more per day)) |
| *Vegetables and fruit* | | | | | | |
| Hoare et al. 2014[26] | multivariable logistic regression | females | did not meet WHO recommendations of 400g/day vs. met WHO recommendations | OR | 1.07 (SE 0.06) | stratified by gender; adjusted for age, parent’s level of education, and school the participant attended |
|  |  | males |  |  | 0.98 (SE 0.09) |  |
| Hoare et al. 2016[31] | cross-sectional analysis: simple logistic regression | females | did not meet WHO recommendations of 400g/day vs. met WHO recommendations | OR | 0.85 (0.54; 1.34) | stratified by gender; no other covariates controlled for in data analysis |
|  |  | males |  |  | 0.87 (0.39; 1.96) |  |
|  | longitudinal analysis: multivariate linear regression | females | did not meet the recommendations at baseline 🡪 did not meet the recommendations at follow up | β | -0.62 (-2.32; 1.07) | stratified by gender; adjusted for school and parental education |
|  |  |  | met the recommendations at baseline 🡪 did not meet the recommendations at follow up |  | -1.2 (-3.31; 0.91) |  |
|  |  |  | did not meet the recommendations at baseline 🡪 met the recommendations at follow up |  | -1.3 (-3.22; 0.62) |  |
|  |  | males | did not meet the recommendations at baseline 🡪 did not meet the recommendations at follow up |  | 0.46 (-0.89; 1.81) |  |
|  |  |  | met the recommendations at baseline 🡪 did not meet the recommendations at follow up |  | -0.55 (-2.03; 0.93) |  |
|  |  |  | did not meet the recommendations at baseline 🡪 met the recommendations at follow up |  | 0.62 (-0.8; 2.04) |  |
| Ming-wei Liu et al.[29] | multivariable logistic regression | Saint Lucia | <5 vs 5 or more servings of fruit and vegetable per day | OR | 0.78 (0.44; 1.38) | Age, sex, physical activity, sedentary behaviour, being bullied, smoking status, alcohol intake |
|  |  | Egypt |  |  | 0.81 (0.58; 1.14) | Age, sex, physical activity, sedentary behaviour, being bullied |
|  |  | Saint Vincent and Grenadines |  |  | 0.85 (0.53; 1.36) | Age, sex, physical activity, sedentary behaviour, being bullied, smoking status, alcohol intake |
|  |  | Djibouti |  |  | 0.88 (0.63; 1.21) | Age, sex, physical activity, sedentary behaviour, being bullied, smoking status |
|  |  | Morocco |  |  | 0.88 (0.72; 1.08) | Age, sex, physical activity, sedentary behaviour, being bullied, smoking status, alcohol intake |
|  |  | Myanmar |  |  | 0.89 (0.46; 1.72) | Age, sex, physical activity, sedentary behaviour, being bullied, smoking status, alcohol intake |
|  |  | Zambia |  |  | 0.94 (0.63; 1.40) | Age, sex, physical activity, sedentary behaviour, being bullied, alcohol intake |
|  |  | United Republic of Tanzania |  |  | 0.97 (0.64; 1.50) | Age, sex, physical activity, sedentary behaviour, being bullied, smoking status, alcohol intake |
|  |  | Venezuela |  |  | 1.00 (0.73; 1.37) | Age, sex, physical activity, sedentary behaviour, being bullied, alcohol intake |
|  |  | Grenada |  |  | 1.01 (0.73; 1.40) | Age, sex, physical activity, sedentary behaviour, being bullied, smoking status, alcohol intake |
|  |  | Lebanon |  |  | 1.03 (0.87; 1.23) | Age, sex, being bullied, alcohol intake |
|  |  | China |  |  | 1.03 (0.87; 1.23) | Age, sex, physical activity, sedentary behaviour, being bullied, smoking status, alcohol intake |
|  |  | Indonesia |  |  | 1.06 (0.87; 1.30) | Age, sex, physical activity, sedentary behaviour, being bullied, smoking status, alcohol intake |
|  |  | Thailand |  |  | 1.07 (0.79; 1.46) | Age, sex, physical activity, sedentary behaviour, being bullied, smoking status, alcohol intake |
|  |  | Uganda |  |  | 1.11 (0.78; 1.58) | Age, sex, physical activity, sedentary behaviour, being bullied, smoking status, alcohol intake |
|  |  | Tunisia |  |  | 1.14 (0.90; 1.44) | Age, sex, physical activity, sedentary behaviour, being bullied, smoking status |
|  |  | Botswana |  |  | 1.15 (0.81; 1.63) | Age, sex, physical activity, sedentary behaviour, being bullied, smoking status, alcohol intake |
|  |  | Sri Lanka |  |  | 1.20 (0.88; 1.64) | Age, sex, physical activity, sedentary behaviour, being bullied |
|  |  | India |  |  | 1.22 (0.95; 1.56) | Age, sex, physical activity, sedentary behaviour, smoking status |
|  |  | Seychelles |  |  | 1.27 (1.18; 1.36) | Age, sex, physical activity, sedentary behaviour, being bullied, smoking status, alcohol intake |
|  |  | Guyana |  |  | 1.31 (0.76; 2.26) | Age, sex, physical activity, sedentary behaviour, being bullied, smoking status, alcohol intake |
|  |  | Ecuador |  |  | 1.39 (1.07; 1.81) | Age, sex, physical activity, sedentary behaviour, being bullied, smoking status, alcohol intake |
|  |  | Jordan |  |  | 1.41 (1.08; 1.83) | Age, sex, physical activity, sedentary behaviour, being bullied, smoking status |
|  |  | Argentina |  |  | 1.46 (0.84; 2.54) | Age, sex, physical activity, sedentary behaviour, being bullied, smoking status, alcohol intake |
|  |  | Kenya |  |  | 1.56 (1.02; 2.38) | Age, sex, physical activity, sedentary behaviour, being bullied, smoking status, alcohol intake |
| Winpenny et al. 2018[32] | cross sectional analysis: multivariable linear regression | total | servings/day | β | -0.40 (-0.71; -0.10) | unadjusted |
|  |  |  |  |  | -0.35 (-0.65; -0.05) | sex, SES |
|  |  |  |  |  | -0.22 (-0.51; 0.08) | sex, SES, smoking level, alcohol consumption, physical activity, sleep |
|  |  |  |  |  | -0.07 (-0.28; 0.14) | stratified by gender; adjusted for sex, SES, smoking level, level of alcohol consumption, PA, sleep, friendship quality, self-esteem, family functioning, percentage body fat, medication use, total energy intake |
|  |  | females |  |  | 0.03 (-0.28; 0.33) |  |
|  |  | males |  |  | -0.19 (-0.47; 0.1) |  |
|  | longitudinal analysis: multivariable linear regression | total |  |  | 0.11 (-0.16; 0.38) | unadjusted |
|  |  |  |  |  | 0.17 (-0.10; 0.45) | sex, SES |
|  |  |  |  |  | 0.16 (-0.12; 0.43) | sex, SES, smoking level, alcohol consumption, physical activity, sleep |
|  |  |  |  |  | 0.14 (-0.15; 0.43) | stratified by gender; adjusted for sex, SES, smoking level, level of alcohol consumption, PA, sleep, friendship quality, self-esteem, family functioning, percentage body fat, medication use, total energy intake, Mood and Feelings Questionnaire score at baseline |
|  |  | females |  |  | 0.21 (-0.22; 0.64) |  |
|  |  | males |  |  | 0.06 (-0.32; 0.44) |  |
| Anxiety | | | | | | |
| *Fruit* | | | | | | |
| Arat 2017[23] | binomial logistic regression | Botswana | high (≥1 time/ day) vs. low (6 times or less during the past 7 days) | OR | 1.1 (1.06; 1.14) | age, gender, bullying, close friends, parental control, hunger (proxy for social status) |
|  |  | Kenya |  |  | 1.28 (1.26; 1.29) |  |
|  |  | Seychelles |  |  | 1.34 (1.07; 1.67) |  |
|  |  | Uganda |  |  | 1.45 (1.41; 1.48) |  |
|  |  | Tanzania |  |  | 0.87 (0.8; 0.94) |  |
|  |  | Zambia |  |  | 1.33 (1.3; 1.36) |  |
| Ming-wei Liu et al.[29] | multivariable logistic regression | Saint Lucia | <2 times/day vs. none | OR | 0.60 (0.27; 1.34) | Age, sex, physical activity, sedentary behaviour, being bullied, smoking status, alcohol intake |
|  |  |  | ≥2 times/day vs. none |  | 0.74 (0.35; 1.54) |  |
|  |  | Egypt | <2 times/day vs. none |  | 0.81 (0.46; 1.43) | Age, sex, physical activity, sedentary behaviour, being bullied |
|  |  |  | ≥2 times/day vs. none |  | 1.25 (0.74; 2.11) |  |
|  |  | Saint Vincent and Grenadines | <2 times/day vs. none |  | 0.88 (0.37; 2.07) | Age, sex, physical activity, sedentary behaviour, being bullied, smoking status, alcohol intake |
|  |  |  | ≥2 times/day vs. none |  | 0.95 (0.39; 2.34) |  |
|  |  | Djibouti | <2 times/day vs. none |  | 0.74 (0.43; 1.28) | Age, sex, physical activity, sedentary behaviour, being bullied, smoking status |
|  |  |  | ≥2 times/day vs. none |  | 0.64 (0.38; 1.07) |  |
|  |  | Morocco | <2 times/day vs. none |  | 0.41 (0.15; 1.11) | Age, sex, physical activity, sedentary behaviour, being bullied, smoking status, alcohol intake |
|  |  |  | ≥2 times/day vs. none |  | 0.34 (0.13; 0.91) |  |
|  |  | Myanmar | <2 times/day vs. none |  | 0.82 (0.26; 2.63) | Age, sex, physical activity, sedentary behaviour, being bullied, smoking status, alcohol intake |
|  |  |  | ≥2 times/day vs. none |  | 0.78 (0.23; 2.60) |  |
|  |  | Zambia | <2 times/day vs. none |  | 1.12 (0.65; 1.93) | Age, sex, physical activity, sedentary behaviour, being bullied, alcohol intake |
|  |  |  | ≥2 times/day vs. none |  | 0.84 (0.49; 1.44) |  |
|  |  | United Republic of Tanzania | <2 times/day vs. none |  | 0.62 (0.22; 1.73) | Age, sex, physical activity, sedentary behaviour, being bullied, smoking status, alcohol intake |
|  |  |  | ≥2 times/day vs. none |  | 0.30 (0.14; 0.65) |  |
|  |  | Venezuela | <2 times/day vs. none |  | 0.55 (0.34; 0.89) | Age, sex, physical activity, sedentary behaviour, being bullied, alcohol intake |
|  |  |  | ≥2 times/day vs. none |  | 0.52 (0.32; 0.84) |  |
|  |  | Grenada | <2 times/day vs. none |  | 1.16 (0.58; 2.30) | Age, sex, physical activity, sedentary behaviour, being bullied, smoking status, alcohol intake |
|  |  |  | ≥2 times/day vs. none |  | 0.96 (0.52; 1.78) |  |
|  |  | Lebanon | <2 times/day vs. none |  | 0.88 (0.51; 1.51) | Age, sex, being bullied, alcohol intake |
|  |  |  | ≥2 times/day vs. none |  | 0.84 (0.52; 1.37) |  |
|  |  | China | <2 times/day vs. none |  | 0.49 (0.34; 0.71) | Age, sex, physical activity, sedentary behaviour, being bullied, smoking status, alcohol intake |
|  |  |  | ≥2 times/day vs. none |  | 0.57 (0.37; 0.87) |  |
|  |  | Indonesia | <2 times/day vs. none |  | 0.53 (0.27; 1.01) | Age, sex, physical activity, sedentary behaviour, being bullied, smoking status, alcohol intake |
|  |  |  | ≥2 times/day vs. none |  | 0.54 (0.31; 0.94) |  |
|  |  | Thailand | <2 times/day vs. none |  | 0.66 (0.39; 1.12) | Age, sex, physical activity, sedentary behaviour, being bullied, smoking status, alcohol intake |
|  |  |  | ≥2 times/day vs. none |  | 0.59 (0.31; 1.11) |  |
|  |  | Uganda | <2 times/day vs. none |  | 0.62 (0.44; 0.87) | Age, sex, physical activity, sedentary behaviour, being bullied, smoking status, alcohol intake |
|  |  |  | ≥2 times/day vs. none |  | 0.63 (0.35; 1.13) |  |
|  |  | Tunisia | <2 times/day vs. none |  | 0.41 (0.27; 0.61) | Age, sex, physical activity, sedentary behaviour, being bullied, smoking status |
|  |  |  | ≥2 times/day vs. none |  | 0.43 (0.31; 0.59) |  |
|  |  | Botswana | <2 times/day vs. none |  | 0.82 (0.55; 1.22) | Age, sex, physical activity, sedentary behaviour, being bullied, smoking status, alcohol intake |
|  |  |  | ≥2 times/day vs. none |  | 0.75 (0.49; 1.14) |  |
|  |  | Sri Lanka | <2 times/day vs. none |  | 0.35 (0.20; 0.62) | Age, sex, physical activity, sedentary behaviour, being bullied |
|  |  |  | ≥2 times/day vs. none |  | 0.43 (0.26; 0.70) |  |
|  |  | India | <2 times/day vs. none |  | 0.54 (0.37; 0.78) | Age, sex, physical activity, sedentary behaviour, smoking status |
|  |  |  | ≥2 times/day vs. none |  | 0.51 (0.34; 0.78) |  |
|  |  | Seychelles | <2 times/day vs. none |  | 0.62 (0.38; 1.004) | Age, sex, physical activity, sedentary behaviour, being bullied, smoking status, alcohol intake |
|  |  |  | ≥2 times/day vs. none |  | 0.58 (0.36; 0.91) |  |
|  |  | Guyana | <2 times/day vs. none |  | 0.55 (0.25; 1.21) | Age, sex, physical activity, sedentary behaviour, being bullied, smoking status, alcohol intake |
|  |  |  | ≥2 times/day vs. none |  | 0.59 (0.24; 1.42) |  |
|  |  | Ecuador | <2 times/day vs. none |  | 0.55 (0.37; 0.80) | Age, sex, physical activity, sedentary behaviour, being bullied, smoking status, alcohol intake |
|  |  |  | ≥2 times/day vs. none |  | 0.61 (0.41; 0.91) |  |
|  |  | Jordan | <2 times/day vs. none |  | 0.59 (0.38; 0.93) | Age, sex, physical activity, sedentary behaviour, being bullied, smoking status |
|  |  |  | ≥2 times/day vs. none |  | 0.60 (0.31; 1.14) |  |
|  |  | Argentina | <2 times/day vs. none |  | 0.35 (0.16; 0.77) | Age, sex, physical activity, sedentary behaviour, being bullied, smoking status, alcohol intake |
|  |  |  | ≥2 times/day vs. none |  | 0.62 (0.28; 1.37) |  |
|  |  | Kenya | <2 times/day vs. none |  | 0.52 (0.36; 0.75) | Age, sex, physical activity, sedentary behaviour, being bullied, smoking status, alcohol intake |
|  |  |  | ≥2 times/day vs. none |  | 0.60 (0.42; 0.86) |  |
| *Vegetables* | | | | | | |
| Arat 2017[23] | binomial logistic regression | Botswana | high (≥1 time/ day) vs. low (6 times or less during the past 7 days) | OR | 1.15 (1.11; 1.2) | age, gender, bullying, close friends, parental control, hunger (proxy for social status) |
|  |  | Kenya |  |  | 0.99 (0.98; 1.01) |  |
|  |  | Seychelles |  |  | 1.88 (1.52; 2.32) |  |
|  |  | Uganda |  |  | 1.58 (1.55; 1.62) |  |
|  |  | Tanzania |  |  | 0.7 (0.64; 0.77) |  |
|  |  | Zambia |  |  | 0.97 (0.94; 0.99) |  |
| Ming-wei Liu et al.[29] | multivariable logistic regression | Saint Lucia | <3 times/day vs. none | OR | 0.72 (0.37; 1.43) | Age, sex, physical activity, sedentary behaviour, being bullied, smoking status, alcohol intake |
|  |  |  | ≥3 times/day vs. none |  | 1.37 (0.62; 3.02) |  |
|  |  | Egypt | <3 times/day vs. none |  | 0.64 (0.36; 1.17) | Age, sex, physical activity, sedentary behaviour, being bullied |
|  |  |  | ≥3 times/day vs. none |  | 0.79 (0.35; 1.80) |  |
|  |  | Saint Vincent and Grenadines | <3 times/day vs. none |  | 0.50 (0.27; 0.92) | Age, sex, physical activity, sedentary behaviour, being bullied, smoking status, alcohol intake |
|  |  |  | ≥3 times/day vs. none |  | 0.88 (0.51; 1.51) |  |
|  |  | Djibouti | <3 times/day vs. none |  | 0.49 (0.27; 0.87) | Age, sex, physical activity, sedentary behaviour, being bullied, smoking status |
|  |  |  | ≥3 times/day vs. none |  | 0.64 (0.32; 1.29) |  |
|  |  | Morocco | <3 times/day vs. none |  | 0.48 (0.22; 1.06) | Age, sex, physical activity, sedentary behaviour, being bullied, smoking status, alcohol intake |
|  |  |  | ≥3 times/day vs. none |  | 0.55 (0.26; 1.15) |  |
|  |  | Myanmar | <3 times/day vs. none |  | 0.42 (0.07; 2.43) | Age, sex, physical activity, sedentary behaviour, being bullied, smoking status, alcohol intake |
|  |  |  | ≥3 times/day vs. none |  | 0.18 (0.03; 1.30) |  |
|  |  | Zambia | <3 times/day vs. none |  | 1.14 (0.75; 1.74) | Age, sex, physical activity, sedentary behaviour, being bullied, alcohol intake |
|  |  |  | ≥3 times/day vs. none |  | 1.45 (0.94; 2.24) |  |
|  |  | United Republic of Tanzania | <3 times/day vs. none |  | 0.57 (0.20; 1.61) | Age, sex, physical activity, sedentary behaviour, being bullied, smoking status, alcohol intake |
|  |  |  | ≥3 times/day vs. none |  | 1.22 (0.57; 2.64) |  |
|  |  | Venezuela | <3 times/day vs. none |  | 0.95 (0.62; 1.44) | Age, sex, physical activity, sedentary behaviour, being bullied, alcohol intake |
|  |  |  | ≥3 times/day vs. none |  | 1.58 (0.88; 2.85) |  |
|  |  | Grenada | <3 times/day vs. none |  | 0.68 (0.34; 1.35) | Age, sex, physical activity, sedentary behaviour, being bullied, smoking status, alcohol intake |
|  |  |  | ≥3 times/day vs. none |  | 0.74 (0.36; 1.52) |  |
|  |  | Lebanon | <3 times/day vs. none |  | 0.49 (0.32; 0.74) | Age, sex, being bullied, alcohol intake |
|  |  |  | ≥3 times/day vs. none |  | 0.57 (0.34; 0.95) |  |
|  |  | China | <3 times/day vs. none |  | 0.29 (0.11; 0.77) | Age, sex, physical activity, sedentary behaviour, being bullied, smoking status, alcohol intake |
|  |  |  | ≥3 times/day vs. none |  | 0.28 (0.10; 0.80) |  |
|  |  | Indonesia | <3 times/day vs. none |  | 1.16 (0.35; 3.82) | Age, sex, physical activity, sedentary behaviour, being bullied, smoking status, alcohol intake |
|  |  |  | ≥3 times/day vs. none |  | 0.99 (0.28; 3.49) |  |
|  |  | Thailand | <3 times/day vs. none |  | 1.59 (0.45; 5.69) | Age, sex, physical activity, sedentary behaviour, being bullied, smoking status, alcohol intake |
|  |  |  | ≥3 times/day vs. none |  | 1.66 (0.49; 5.67) |  |
|  |  | Uganda | <3 times/day vs. none |  | 0.71 (0.48; 1.04) | Age, sex, physical activity, sedentary behaviour, being bullied, smoking status, alcohol intake |
|  |  |  | ≥3 times/day vs. none |  | 1.11 (0.60; 2.05) |  |
|  |  | Tunisia | <3 times/day vs. none |  | 0.74 (0.46; 1.19) | Age, sex, physical activity, sedentary behaviour, being bullied, smoking status |
|  |  |  | ≥3 times/day vs. none |  | 0.94 (0.56; 1.59) |  |
|  |  | Botswana | <3 times/day vs. none |  | 0.81 (0.47; 1.41) | Age, sex, physical activity, sedentary behaviour, being bullied, smoking status, alcohol intake |
|  |  |  | ≥3 times/day vs. none |  | 1.13 (0.61; 2.09) |  |
|  |  | Sri Lanka | <3 times/day vs. none |  | 0.61 (0.15; 2.45) | Age, sex, physical activity, sedentary behaviour, being bullied |
|  |  |  | ≥3 times/day vs. none |  | 0.69 (0.20; 2.36) |  |
|  |  | India | <3 times/day vs. none |  | 1.06 (0.55; 2.05) | Age, sex, physical activity, sedentary behaviour, smoking status |
|  |  |  | ≥3 times/day vs. none |  | 1.07 (0.52; 2.20) |  |
|  |  | Seychelles | <3 times/day vs. none |  | 0.58 (0.41; 0.82) | Age, sex, physical activity, sedentary behaviour, being bullied, smoking status, alcohol intake |
|  |  |  | ≥3 times/day vs. none |  | 0.60 (0.42; 0.86) |  |
|  |  | Guyana | <3 times/day vs. none |  | 0.79 (0.30; 1.12) | Age, sex, physical activity, sedentary behaviour, being bullied, smoking status, alcohol intake |
|  |  |  | ≥3 times/day vs. none |  | 1.36 (0.43; 4.27) |  |
|  |  | Ecuador | <3 times/day vs. none |  | 0.61 (0.42; 0.88) | Age, sex, physical activity, sedentary behaviour, being bullied, smoking status, alcohol intake |
|  |  |  | ≥3 times/day vs. none |  | 0.70 (0.48; 1.002) |  |
|  |  | Jordan | <3 times/day vs. none |  | 0.86 (0.37; 1.97) | Age, sex, physical activity, sedentary behaviour, being bullied, smoking status |
|  |  |  | ≥3 times/day vs. none |  | 0.67 (0.25; 1.82) |  |
|  |  | Argentina | <3 times/day vs. none |  | 0.97 (0.54; 1.76) | Age, sex, physical activity, sedentary behaviour, being bullied, smoking status, alcohol intake |
|  |  |  | ≥3 times/day vs. none |  | 0.41 (0.15; 1.16) |  |
|  |  | Kenya | <3 times/day vs. none |  | 0.94 (0.47; 1.88) | Age, sex, physical activity, sedentary behaviour, being bullied, smoking status, alcohol intake |
|  |  |  | ≥3 times/day vs. none |  | 1.12 (0.56; 2.27) |  |
| Depression and anxiety | | | | | | |
| *Vegetables and fruit* | | | | | | |
| McMartin et al. 2012[33] | negative binomial regression | N/A | 2^nd^ tertile vs 1^st^ tertile | IRR | 1.06 (0.68; 1.66) | energy intake |
|  |  |  | 3^rd^ tertile vs 1^st^ tertile |  | 1.15 (0.73; 1.81) |  |
|  |  |  | 2^nd^ tertile vs 1^st^ tertile |  | 1.04 (0.71; 1.53) | energy intake, gender, household income, parental marital status and education, body weight status, PA and geographic area |
|  |  |  | 3^rd^ tertile vs 1^st^ tertile |  | 1.25 (0.8; 1,99) |  |

**Supplementary File 4**

**PRISMA-S Checklist**

| **INFORMATION SOURCES AND METHODS** | | | |
| --- | --- | --- | --- |
| Database name | 1 | Name each individual database searched, stating the platform for each. | 1 |
| Multi-database searching | 2 | If databases were searched simultaneously on a single platform, state the name of the platform, listing all of the databases searched. (Cochrane Library (Reviews and Trials) | 1 |
| Study registries | 3 | List any study registries searched. | 2 (e.g., PROSPERO) |
| Online resources and browsing | 4 | Describe any online or print source purposefully searched or browsed (e.g., tables of contents, print conference proceedings, web sites), and how this was done. | We pilot searched several relevant websites, however, our search did not yield any relevant publications. |
| Citation searching | 5 | Indicate whether cited references or citing references were examined, and describe any methods used for locating cited/citing references (e.g., browsing reference lists, using a citation index, setting up email alerts for references citing included studies). | 1-2 |
| Contacts | 6 | Indicate whether additional studies or data were sought by contacting authors, experts, manufacturers, or others. | 2 |
| Other methods | 7 | Describe any additional information sources or search methods used. | N/A |
| **SEARCH STRATEGIES** | | | |
| Full search strategies | 8 | Include the search strategies for each database and information source, copied and pasted exactly as run. | Suppl File 1 |
| Limits and restrictions | 9 | Specify that no limits were used, or describe any limits or restrictions applied to a search (e.g., date or time period, language, study design) and provide justification for their use. | 1-2 |
| Search filters | 10 | Indicate whether published search filters were used (as originally designed or modified), and if so, cite the filter(s) used. | Suppl File 1 |
| Prior work | 11 | Indicate when search strategies from other literature reviews were adapted or reused for a substantive part or all of the search, citing the previous review(s). | N/A |
| Updates | 12 | Report the methods used to update the search(es) (e.g., rerunning searches, email alerts). | 1-2 |
| Dates of searches | 13 | For each search strategy, provide the date when the last search occurred. | Suppl File 1 |
| **PEER REVIEW** | | | |
| Peer review | 14 | Describe any search peer review process. | N/A |
| **MANAGING RECORDS** | | | |
| Total Records | 15 | Document the total number of records identified from each database and other information sources. | Suppl File 1 |
| Deduplication | 16 | Describe the processes and any software used to deduplicate records from multiple database searches and other information sources. | 2 |
|  |  |  |  |
| PRISMA-S: An Extension to the PRISMA Statement for Reporting Literature Searches in Systematic Reviews | | |  |
| Rethlefsen ML, Kirtley S, Waffenschmidt S, Ayala AP, Moher D, Page MJ, Koffel JB, PRISMA-S Group. | | |  |
| Last updated February 27, 2020. | |  |  |
